# Antibiotic Prescription for COPD Exacerbations Admitted to Hospital: European COPD Audit

**DOI:** 10.1371/journal.pone.0124374

**Published:** 2015-04-23

**Authors:** Jose Luis López-Campos, Sylvia Hartl, Francisco Pozo-Rodriguez, C. Michael Roberts, on behalf of the European COPD Audit team

**Affiliations:** 1 Hospital Universitario Virgen del Rocío, Instituto de Biomedicina de Sevilla (IBiS), Seville, Spain; 2 CIBER de Enfermedades Respiratorias (CIBERES), Instituto de Salud Carlos III, Madrid, Spain; 3 Ludwig Boltzmann Institute of COPD and Respiratory Epidemiology, Vienna, Austria; 4 Hospital 12 de Octubre, Instituto de Investigación i+12, Madrid, Spain; 5 Barts and The London School of Medicine and Dentistry, Queen Mary, University of London, United Kingdom; University of Athens Medical School, GREECE

## Abstract

**Objective:**

Appropriate use of antibiotics in the management of hospitalised patients with COPD exacerbations is defined within the GOLD strategy. This paper analyses the factors associated with antibiotic prescribing in patients to better understand how prescribing may be improved.

**Methods:**

The European COPD audit was a study of clinical care in 384 hospitals from 13 European countries between 2010 and 2011 enrolling 16018 patients. Those admitted to hospital due to a clinician-made diagnosis of exacerbation of COPD at the time of discharge were audited. We defined antibiotic prescribing compliance as consistent with the GOLD 2010 recommendations. Two different multivariate models were used to evaluate factors associated with the prescription of antibiotics and the guideline-compliant prescriptions.

**Results:**

Overall 86% of admissions were given antibiotics but only 61.4% cases met the GOLD recommendations. Antibiotics were more likely to be given in hospital and at discharge if received prior to admission. Antibiotic prescription was more likely in patients who met the GOLD recommendations and in those with radiological consolidation but there was also a significant use of antibiotics in patients who did not meet either criterion. Patients cared for on a Respiratory Ward were more likely to receive GOLD compliant antibiotic management.

**Conclusions:**

The present study describes the audited in-hospital antibiotic prescription for COPD exacerbation across different European countries. In general, there is an apparent overuse of antibiotics likely to be associated with both patient and practice-related variables.

## Introduction

Although antibiotic prescription is one of the mainstay treatments for exacerbations of chronic obstructive pulmonary disease, a number of controversies are associated with the use of this therapy. It is widely acknowledged that antibiotics should not be prescribed for all exacerbations, but only to those caused by a bacterial infection [1, 2]. The problem arises when there are no rapid culture tests than can confirm that a particular exacerbation is caused by a bacterial infection in the acute setting. Whilst alternative approaches have been proposed and several biomarkers have shown promise in this regard, [3] to date there is no universally accepted test to guide initial clinical decision making at the point of hospital presentation [4, 5]. As a result, the decision to prescribe antibiotics for exacerbation of chronic obstructive pulmonary disease (COPD) is currently based on clinical criteria, led mainly by the presence of purulent sputum [2, 6]. The Global Initiative for Obstructive Lung Disease (GOLD) strategy document makes evidenced recommendations for antibiotic indication in hospitalised patients admitted for management of COPD exacerbations [4] derived from the European guidelines for the management of lower respiratory tract infections [7].

The evidence suggests that the use of antibiotics in these patient groups is associated with more rapid recovery times and reduced short term mortality [8]. However, a recent Cochrane review showed inconsistent results for outpatients and inpatients not requiring intensive care unit admission [9]. In contrast the use of antibiotics in patients who do not meet these criteria may cause unjustified side effects, increases the risk of emerging microbial resistance and adds unnecessary cost to an admission[10].

The European COPD Audit was a pilot study funded by the European Respiratory Society carried out in 384 hospitals from 13 European countries between 2010 and 2011, which collected COPD admissions data on 16018 exacerbations of COPD in order to investigate adherence to the GOLD recommendations for COPD management across Europe [11]. During this European audit the prescription of antibiotics was recorded in all included cases. The present paper aims to analyse the European COPD audit database to explore which factors are associated with antibiotic prescription and specifically the correct use of antibiotics.

## Methods

This analysis is based on a secondary analysis of the European COPD Audit. The methodology of the audit has been reported in detail previously [11]. Briefly, clinical process and outcomes indicators for consecutive admissions were assessed over an 8-week period with a second round of data collection for each admission at 90 days to review late outcomes. Eligibility for inclusion in the audit was a clinician-made diagnosis of exacerbation of COPD at the point of admission which was then confirmed by a senior clinician at the time of discharge from hospital. Patients with a primary diagnosis other than exacerbation (e.g. clinical pneumonia) at discharge were withdrawn from the study cohort. Information on demographics, previous history, clinical data upon admission, data during admission and information on discharge and follow up, as well as the resources of the participant centre were collected on a webtool (IDCode, Lausanne Switzerland) [11]. The European audit followed the European ethical requirements for scientific studies. All partners of the project accepted the general ethical rules of the ERS, particularly the rules on conflict of interests and relationships with the tobacco industry. As there is no European Ethics Committee for audits, national societies ensured compliance with European and national ethical requirements. In addition, the project was approved by the Ethics Committee from Hospital Universitario Virgen del Rocío (approval acta 16/2010). Since this was a retrospective non-interventional study, patient informed consent was deemed not to be necessary. Patient records and information were anonymized and de-identified prior to analysis.

The GOLD strategy update 2010, based on the guidelines for the management of lower respiratory tract infections [7], current at the time of the audit, recommended that for hospital care antibiotics should be given to patients who presented with an increase in all three of the following cardinal symptoms: dyspnea, sputum volume, and sputum purulence, patients with two of the cardinal symptoms if increased purulence of sputum is one of the two symptoms, and patients who require mechanical ventilation (invasive or non-invasive). Those cases with these indications and an antibiotic prescription and those cases not fulfilling this indication and not receiving antibiotics were considered to have guideline compliant use of antibiotics. Consequently, GOLD compliance was defined as the proportion of patients prescribed antibiotics who met the GOLD recommendations plus the proportion of patients who did not receive antibiotics and who did not meet them. The precision of antibiotic prescribing was defined as the proportion of patients given antibiotics who met the GOLD recommendations. In the present paper, factors associated with prescription and GOLD compliant prescription were analysed in two separate models. In a further analysis patients with reported radiological consolidation were added to those who met the GOLD recommendations as compliant with appropriate antibiotic prescribing.

### Statistics

The statistical computations were performed with the Statistical Package for Social Sciences (SPSS, IBM Corporation, Somers, NY) version 20.0. Descriptive data expressed as mean (standard deviation) and absolute (relative) frequencies depending on the nature of the variable and the inter-country range, indicating those countries with the lowest and highest values for each item. To give an indication of the variability of the variables we calculated the inter-country range (ICR), which indicates the country with the highest or lowest mean value for a particular variable. An initial analysis to investigate factors associated with the prescription of antibiotics (regardless of if it was an appropriate prescription or not) and a second to investigate factors associated with the correct prescription of antibiotics. In both analyses bivariate comparisons were performed using the unpaired T test (using Levene's test to assess the equality of variances) or the chi-squared test comparing both groups. To evaluate those factors associated with antibiotic prescription two binomial multivariate logistic regression analyses were performed. Those variables significant in the bivariate analysis were included in the multivariate analyses as explanatory variables. The dependent variables were the prescription of antibiotics and the correct prescription of antibiotics respectively. Differences between groups were analysed by using the log-rank test. The alpha error was set at 0.05.

## Results

**Fig 1:**
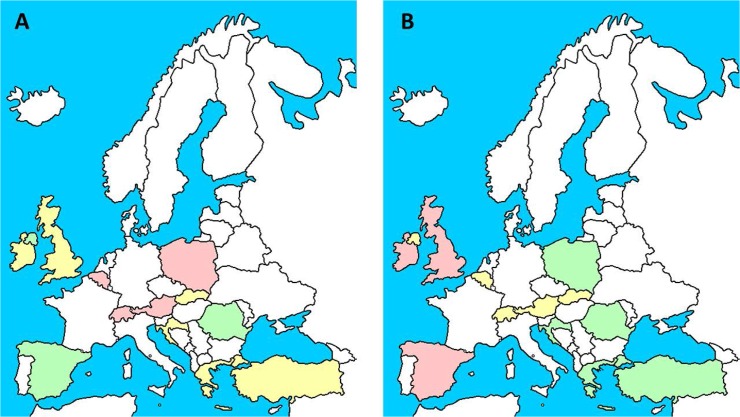
Distribution of antibiotic prescription (A) and correct antibiotic prescription (B) between the participant countries. Red represent those countries with an average value significantly lower than the European average. Yellow represent those countries not significantly different from the European average. Green represent those countries with an average value significantly higher than the European average. White countries did not participate in the audit. Figure for illustrative purposes only.

The number of patients included in the study was 16018. All patients had valid information on whether they were receiving antibiotics or not with no missing values. However, when analysing the prescription by the presenting symptoms or mechanical ventilation in 65 (0.4%) cases there was insufficient information to determine if GOLD compliance had been achieved or not. The sample size was therefore 16018 for analysing antibiotic prescription, and 15953 for analysing GOLD compliance prescription. The distribution of antibiotic and guideline compliant antibiotic prescription in the different participant countries is documented in Table 1 and depicted in Fig 1. The number of cases prescribed antibiotics was 13773 (86.0%) ranging from 54.3% in Austria to 95.4% in Ireland. The number of cases with guideline compliant prescription was 9777 (61.3%) ranging from 58.2% in Malta to 68.1% in Poland and precision of prescribing for patients given antibiotics ranging from 55.3% for Malta to 66.9% in Turkey increasing to 74.8% including consolidation as an indication.

**Table 1:** Distribution of antibiotic prescription in every participant country considering the GOLD criteria or the GOLD criteria plus the presence of consolidation.

Country	Antibiotic prescription¥ (n = 16018)	GOLD criteria		GOLD + consolidation criteria	
		Correct antibiotic prescription† ¥ (n = 15953)	Precision of prescribing ‡ § (n = 13773)	Correct antibiotic prescription† ¥ ¶ (n = 15961)	Precision of prescribing ‡ § ¶ (n = 13773)
Austria (n = 823)	447 (54.3)*	496 (60.3)	267 (59.7)	512 (62.3) *	305 (68.2)
Belgium (n = 513)	388 (75.6) *	327 (63.7)	240 (61.9)	360 (70.2)	275 (70.9)
Croatia (n = 374)	374 (84.0)	297 (66.7) *	256 (68.4) *	320 (71.9) *	282 (75.4) *
Greece (n = 1133)	988 (87.2)	729 (64.7) *	668 (67.6) *	783 (69.4) *	732 (74.1) *
Ireland (n = 237)	226 (95.4) *	158 (66.7)	152 (67.3)	174 (73.4) *	168 (74.3)
Malta (n = 112)	105 (93.8) *	64 (58.2)	57 (55.3)	69 (62.7)	62 (59.0) *
Poland (n = 734)	525 (71.5) *	496 (68.1) *	353 (68.0) *	524 (71.9) *	400 (76.2) *
Romania (n = 629)	587 (93.3) *	411 (65.3) *	396 (67.5) *	462 (73.4) *	451 (76.8) *
Slovakia (n = 42)	36 (85.7)	25 (59.5)	22 (61.1)	27 (64.3)	25 (69.4)
Spain (n = 5271)	4888 (92.7) *	3122 (59.6) *	2876 (59.3) *	3284 (62.7) *	3046 (62.3) *
Switzerland (n = 295)	214 (72.5) *	169 (60.7)	136 (63.6)	193 (65.4)	157 (73.4)
Turkey (n = 612)	536 (87.6)	401 (65.6) *	374 (69.9) *	422 (69.1)	401 (74.8) *
United Kingdom (n = 5172)	4459 (86.2)	3072 (59.5) *	2633 (59.2) *	3463 (67.1)	3048 (68.4) *
Total (n = 16018)	13773 (86.0)	9777 (61.3)	8430 (61.4)	10593 (66.4)	9352 (67.9)

Data expressed in absolute (relative) frequencies.

* p < 0.05 against the rest of the countries.

† Refers to the correct prescription as those with indication and prescription plus those with no indication and no prescription.

‡ Refers to the number of patients with a correct prescription among those with an antibiotic prescription.

¥ Percentages referred to the total number of patients in each row.

§ Percentages referred to the total number of patients in the antibiotic prescription column.

¶ Comparison versus GOLD criteria alone significant for the whole cohort and every country.

The characteristics of the patients included is summarised in Table 2. The majority were men, in their seventies, with a significant tobacco history and around 30% were active smokers, with a considerable number of comorbidities, and a predominance of severe and very severe spirometric impairment. The results of the bivariate analysis comparing the variables related to the exacerbation severity, treatments before and during the admission at discharge, and the adjustment to GOLD guidance are summarised in the Table 3 and S1 Table, S2 Table, and S3 Table. All significant associations were considered for the multivariate analysis.

**Table 2:** Characteristics of the patients included.

	Average (n = 16018)	ICR
Age (years)	70.7 (10.7)	63.4–72.7
Male gender (n)	10865 (67.8)	51.9–86.0
Current smokers (n)	5012 (31.3)	15.4–46.8
Never smokers (n)	852 (5.3)	0–18.9
Tobacco history (pack-years)	47.2 (31.2)	29.8–70.8
Comorbidities (Charlson)	2.3 (1.6)	1.7–3.1
Cardiovascular diseases (n)	6491 (40.5)	33.9–71.4
Diabetes (n)	3138 (19.9)	11.9–27.5
Neoplasms (n)	1950 (12.2)	2.6–18.9
Body mass index (kg/m^2^)	26.6 (6.4)	25.0–28.5
Admissions the previous year (n)	1.2 (1.8)	0.4–1.8
Two or more admissions in the previous year (n)	4236 (28.9)	11.1–42.9
Spirometry: FVC (%)	65.4 (20.3)	54.2–74.9
Spirometry: FEV_1_ (%)	44.0 (17.4)	38.9–56.1
Spirometric classification:		
• No spirometry	6512 (40.7)	9.9–53.6
• No obstruction	1226 (7.7)	3.2–34.0
• FEV_1_ > 80%	195 (1.2)	0–3.0
• FEV_1_ 50–80%	2175 (13.6)	2.7–31.0
• FEV_1_ 30–50%	3737 (23.3)	11.6–34.9
• FEV_1_ < 30%	2098 (13.1)	4.8–23.8
• FEV_1_ missing	75 (0.5)	0–2.7

Data expressed as mean (standard deviation) and absolute (relative) frequencies depending on the nature of the variable. ICR: inter-country range.

**Table 3:** Characteristics of the patients included in the study between the study groups.

	No prescription (n = 2245)	Prescription (n = 13773)	P value*	Not correct (n = 6176)	Correct (n = 9777)	P value*
Age (years)	69.6 (10.6)	70.9 (10.7)	< 0.001	71.5 (10.8)	70.2 (10.6)	< 0.001
Male gender (n)	1376 (61.3)	9489 (68.9)	< 0.001	4115 (66.6)	6703 (68.6)	0.011
Current smokers (n)	749 (33.4)	4263 (31.0)	0.002	1938 (31.4)	3060 (31.3)	< 0.001
Tobacco history (pack-years)	43.5 (29.6)	47.8 (31.4)	< 0.001	46.5 (30.2)	47.7 (31.8)	0.046
Comorbidities (Charlson)	2.4 (1.6)	2.36 (1.5)	0.322	2.36 (1.5)	2.37 (1.6)	0.531
Cardiovascular diseases (n)	959 (42.7)	5532 (40.2)	0.023	2528 (40.9)	3933 (40.2)	0.380
Diabetes (n)	469 (20.9)	2712 (19.7)	0.189	1211 (19.6)	1956 (20.0)	0.541
Neoplasms (n)	245 (10.9)	1705 (12.4)	0.052	741 (12.0)	1199 (12.3)	0.636
Body mass index (kg/m^2^)	26.7 (12.7)	26.6 (6.3)	0.629	26.5 (6.2)	26.6 (6.5)	0.527
Admissions the previous year (n)	1.2 (1.9)	1.2 (1.8)	0.377	1.13 (1.7)	1.27 (1.8)	< 0.001
Two or more admissions in the previous year (n)	564 (28.5)	3672 (29.0)	0.631	1498 (24.3)	2724 (27.9)	< 0.001
Spirometry: FVC (%)	65.8 (20.7)	65.4 (20.3)	0.508	66.4 (20.6)	64.8 (20.2)	< 0.001
Spirometry: FEV_1_ (%)	45.6 (18.3)	43.7 (17.3)	0.001	45.2 (17.4)	43.3 (17.4)	< 0.001
Spirometric classification:						
• No spirometry	928 (41.3)	5584 (40.5)	0.487	2598 (42.1)	3883 (39.7)	0.003
• No obstruction	220 (9.8)	1006 (7.3)	< 0.001	472 (7.6)	748 (7.7)	0.999
• FEV_1_ > 80%	30 (1.3)	165 (1.2)	0.610	79 (1.3)	116 (1.2)	0.607
• FEV_1_ 50–80%	302 (13.5)	1873 (13.6)	0.866	890 (14.4)	1280 (13.1)	0.019
• FEV_1_ 30–50%	487 (21.7)	3250 (23.6)	0.050	1406 (22.8)	2317 (23.7)	0.179
• FEV_1_ < 30%	266 (11.8)	1832 (13.3)	0.060	706 (11.4)	1383 (14.1)	< 0.001
• FEV_1_ missing	12 (0.5)	63 (0.5)	0.616	25 (0.4)	50 (0.5)	0.405

Data expressed as mean (standard deviation) and absolute (relative) frequencies depending on the nature of the variable.

* p value calculated by Chi-squared test or Student T test for independent variables as appropriate.

The results of the multivariate analysis indicating those factors associated with the prescription of antibiotics during the admission are summarised in Table 4. There were some predictable associations relating to the GOLD indications but also radiographic consolidation (89.6% of cases received antibiotics) and treatment with antibiotics before the admission (90.1% cases received inpatient antibiotics). There were also associations with other treatments such as systemic steroids, oxygen therapy and non-invasive ventilation, suggesting that antibiotic use was associated with more intense treatment generally. Patients receiving antibiotics before the admission also had a higher probability to continue receiving antibiotics during and after the admission. In contrast the severity of the exacerbation as evaluated by the pH or the severity of the underlying COPD were not associated with likelihood of receiving antibiotics and female gender with lower probability of receiving antibiotics during the admission

**Table 4:** Multivariate analysis of variables associated with the prescription of antibiotics.

	Crude			Adjusted		
	Odds ratio	95% CI Lower limit	95% CI Upper limit	Odds ratio	95% CI Lower limit	95% CI Upper limit
Female gender	0.715	0.652	0.784	0.617	0.617	0.892
Sputum increase	2.553	2.324	2.804	1.350	1.350	2.103
Sputum colour change	2.679	2.428	2.955	1.401	1.401	2.200
Radiology: bronchiectasis	1.979	1.536	2.550	2.319	1.380	3.898
Radiology. consolidation	2.354	2.030	2.728	1.966	1.489	2.597
Treatments before admission: antibiotics	1.605	1.416	1.820	1.527	1.183	1.971
Treatments during admission: short-acting bronchodilators	1.848	1.686	2.027	1.600	1.319	1.940
Treatments during admission: inhaled steroids	0.861	0.785	0.945	0.801	0.670	0.959
Treatments during admission: systemic steroids	2.237	2.021	2.477	2.223	1.754	2.817
Treatments during admission: oxygen	2.468	2.212	2.754	2.530	1.963	3.261
Treatments during admission: non-invasive ventilation	1.382	1.196	1.597	1.340	1.032	1.741
Treatments at discharge: systemic steroids	1.177	1.077	1.287	0.667	0.550	0.809
Treatments at discharge: antibiotics	6.046	5.311	6.882	7.268	5.631	9.380

The results of the multivariate analysis indicating those factors associated with guideline compliant prescription of antibiotics during the admission is summarised in Table 5. Again, there were some expected variables associated including changes in sputum increase or colour or mechanical ventilation, since these are the recommended indications. However, on this occasion the severity of the exacerbation as measured by the pH and PaCO_2_, and being managed on a respiratory ward was also associated with better guideline compliant prescription of antibiotics during the admission. Similarly, there were associations of correct prescription of antibiotics with several other treatments, but not with the use of antibiotics prior to the admission and compliance was associated with lower likelihood of receiving discharge antibiotics. Finally, compliant antibiotic prescription was also associated with receipt of domiciliary therapies (i.e. home mechanical ventilation and home oxygen therapy).

**Table 5:** Multivariate analysis of variables associated with the guideline-compliant prescription of antibiotics.

	Crude			Adjusted		
	Odds ratio	95% CI Lower limit	95% CI Upper limit	Odds ratio	95% CI Lower limit	95% CI Upper limit
Respiratory ward	1.328	1.161	1.321	1.217	1.014	1.460
Dyspnoea increase	1.621	1.349	1.948	2.186	1.268	3.771
Sputum increase	4.272	3.972	4.594	0.660	0.533	0.816
Sputum colour change	23.084	21.041	25.325	52.323	41.563	65.869
pH	0.048	0.029	0.079	0.093	0.015	0.559
PaCO2	1.132	1.112	1.153	0.913	0.854	0.977
Treatments before admission: inhaled steroids	1.207	1.127	1.292	1.196	0.985	1.453
Treatments before admission: methylxanthines	1.203	1.102	1.313	1.324	1.052	1.666
Treatments during admission: systemic steroids	1.148	1.057	1.248	1.401	1.102	1.782
Treatments during admission: methylxanthines	1.266	1.153	1.389	0.749	0.584	0.960
Treatments during admission: oxygen	1.209	1.102	1.325	0.649	0.480	0.877
Treatments during admission: non-invasive ventilation	6.244	5.426	7.187	14.598	10.034	21.237
Treatments during admission: invasive ventilation	7.417	4.837	11.372	8.724	3.876	19.635
Treatments at discharge: antibiotics	0.920	0.862	0.981	0.745	0.625	0.887
Treatments at discharge: oxygen	1.328	1.239	1.423	1.256	1.043	1.512
Treatments at discharge: home mechanical ventilation	2.986	2.491	3.579	0.666	0.427	1.037

In some cases who did not meet the criteria for antibiotic therapy radiological consolidation was recorded. These cases were judged to be appropriate for antibiotic therapy and when included in the compliance and precision calculations increased both by 5.1% and 6.5% respectively. Nevertheless compliance was still only 66.4% and precision in those receiving antibiotics just 67.9%.

## Discussion

The present analysis of the European COPD Audit indicates that the prescription of antibiotics shows some variability among the participant countries and centres and indicates which variables are associated with the prescription and the correct prescription of antibiotics during admission. Overall a very high proportion of patients (86%) received antibiotics and a much lower proportion (61.3%) were managed appropriately either with or without antibiotics according to the GOLD recommendations.

Despite some trials suggesting that the use of antibiotics in exacerbations is associated with a reduced short term mortality [8], other trials have not found an effect [12, 13]. A seminal Cochrane review showed inconsistent results for outpatients and inpatients not requiring intensive care unit admission [9]. However, new studies published after the audit have provided evidence that demonstrate the efficacy of antibiotics in bacterial exacerbations related to antibiotic choice [14, 15]

We found at national level wide variations between the prevalence of antibiotic use ranging from 54.3% in Austria to 92.7% in Spain and 95.4% in Ireland. In the Austrian cohort compliance was 60.3%, in Spain compliance was similar at 59.6% and in Ireland higher at 66.7%. This variance is similar to that of a cross-sectional European study in primary care carried out in January and February 2008 in six countries (Denmark, Sweden, Lithuania, Russia, Spain and Argentina) which registered all patients with exacerbations during a 3-week period. A total of 617 GPs treated 1233 patients with exacerbations. 970 patients (79%) were prescribed antibiotics, varying from 49% (Denmark) to 93% (Russia).[6]

Our study found that particularly antibiotic prescription was associated with antibiotic treatment started prior to admission. Of concern is that this group of patients was also more likely to have antibiotics continued post discharge. Receipt of antibiotics post discharge was not however associated with compliant prescribing, further suggesting inappropriate continuity of use. Clinicians should challenge the need to continue antibiotics in such cases and not automatically prescribe at admission. Antibiotics were also given as part of a management package which included steroids, oxygen and NIV to patients presumably reflecting a tendency to give ‘all treatments’ to patients regarded as ‘sick’.

The main issue here however was prescribing for patients who did not meet the indications. In general just over a half to two thirds of patients within participating countries received antibiotic management consistent with the guidelines. Our results indicate that the presence of consolidation on the chest radiography was a predictor of antibiotic prescription. Whilst there is global agreement that chest consolidation should be considered as pneumonia, COPD exacerbations often present with small transient radiological consolidations not considered as pneumonia by senior managing clinicians[16]. Whilst there remains some debate about the significance of incidental consolidation in COPD exacerbation[17, 18], exemplified by the increasingly frequent reporting on CT pulmonary angiograms, we performed a second analysis including this as a justifiable additional criterion for antibiotic prescription. Future guidelines may make specific reference to this situation. This inclusion within our study raised compliance and precision rates but still one third of admissions were not managed even according to these extended indications and only two thirds of antibiotic prescriptions given met the agreed criteria.

Among the factors associated with the prescription of antibiotics, gender deserves a comment. Although reports regarding gender-related differences in COPD expression have provided conflicting results, several studies indicate that COPD has a different clinical presentation and burden of the disease in women as compared to men [19, 20]. In this regard, women have been described to have more pronounced dyspnoea and lower body mass index, suggesting worse prognosis, and were more likely to exhibit anxiety [21]. These findings suggests that women may need specific assessment and care related to the clinical expression of the disease, but does not justify the under-prescription of antibiotics in women as compared with men described in the present paper. We acknowledge that this could be a statistical finding, but these results deserve further debate and future research in different cohorts, since it may raise a broader issue that should be taken into consideration. Rather than under prescribing, this may represent more precise prescribing in women, as they are no less likely to receive appropriate antibiotics than are men.

Previous use of inhaled steroids was not associated with the use of antibiotics. This is striking especially in a large database like this, in which small differences may produce a significant p value due to the sample size. The use of inhaled corticosteroids in patients with COPD has been associated with an increased incidence of respiratory infections[22]. Although the aim of the present analysis was not to evaluate a potential association between inhaled steroids and infection, it is of note the lack of relationship with antibiotic prescription after considering all the confounders in the multivariate analysis.

Another interesting finding is the association of the respiratory ward in relation to a correct antibiotic prescription. This finding has not been previously described in national audits. In the AUDIPOC study, no consistent differences between respiratory and non-respiratory wards were detected in Spain [23].

Several considerations need to be taken into account to correctly interpret our results. The European COPD was a pilot study aiming at evaluating health care across Europe. Since this was a pilot study, the number of variables collected was intentionally limited to make the work accomplishable. As a consequence, many other unrecorded variables could further explain part of the variability observed, including the microbiology or the drug adverse effects. This paper describes European practice in the context of clinically diagnosed COPD as spirometric confirmation of diagnosis was unavailable in a significant number of cases. The reporting of antibiotic use is dependent upon centres providing accurate and honest data. Antibiotic use was not however a primary endpoint of the audit and the data collected demonstrates such variability both within and across countries that it is most unlikely that there is widespread bias in the reporting. We used GOLD recommendations derived from European guidelines for the management of adult respiratory infections in hospitalised COPD patients to define a correct indication for prescription [7], but other guidelines may have different recommendations. In addition, there may be other possible reasons for prescribing antibiotics that would be appropriate clinically and not recorded as a recommendation in the GOLD strategy document. For instance, there is a strong association between bronchiectasis and antibiotic prescription (Table 2). Although the presence of bronchiectasis is not included in the GOLD recommendations, many would argue that exacerbations in bronchiectasis should always receive antibiotics [24]. It is also the case that we did not have information regarding the type of antibiotic, dose or detailed duration, all factors that are important in determining if a prescription even when indicated is ‘correct’. Finally, the selection of countries and hospitals was based on voluntary participation, so there is no intention of a random sample or of a controlled trial. Regretfully, Scandinavia and central Europe including the biggest member states have not taken part in the audit. The possible effects this could have on the results are unknown. The complexity of recording data through different health-care systems and from different information sources was also challenging for accuracy yet the information recorded is deemed to reflect real-life medicine and is the most valid information available.

In conclusion, the present study analyses the factors associated with the prescription of antibiotics during an admission due to exacerbation of COPD with a special emphasis on the variables associated with the correct prescriptions. The information provided in the present study suggests improvements could be made in both prescribing for patients who meet appropriate criteria and not prescribing for those who have no evidence based indication. Overall there is an over-prescription of antibiotics with likely impacts on costs, morbidity, length of stay, and risk of resistance to the greater community. Managing patients on respiratory specialist wards may provide an environment that promotes better prescribing compliance. There remains an urgent need to provide better evidence for the role of antibiotics in COPD exacerbations admitted to hospital and define which populations benefit from this treatment.

## Supporting Information

S1 Table Exacerbation-related variables reflecting the clinical presentation or the severity of the exacerbation between the study groups. (DOCX)

S2 Table Management-related variables between the study groups. (DOCX)

S3 Table Guidelines compliance between the study groups. (DOCX)

S1 Text European COPD Audit list of investigators. (DOC)
