# Supplementary material for: Antibiotic Prescription for COPD Exacerbations Admitted to Hospital: European COPD Audit
Source: PLoS One. 2015 Apr 23;10(4):e0124374. doi: 10.1371/journal.pone.0124374 (PMC4408103; doi:10.1371/journal.pone.0124374)
Supplement: S1 Table — (DOCX) [file pone.0124374.s001.docx]

**S1 Table. Exacerbation-related variables reflecting the clinical presentation or the severity of the exacerbation between the study groups.**

|  | No antibiot  (n=2245) | Antibiot  (n=13773) | P value* | Not correct  (n=6176) | Correct  (n=9777) | P value* |
| --- | --- | --- | --- | --- | --- | --- |
| Dyspnoea increase (n) | 2151 (96.8) | 13258 (97.1) | 0.461 | 5874 (95.1) | 9481 (97.0) | < 0.001 |
| Sputum increase (n) | 1032 (49.7) | 9341 (71.6) | < 0.001 | 2795 (45.3) | 7555 (77.3) | < 0.001 |
| Sputum colour change (n) | 704 (34.9) | 7430 (59.0) | < 0.001 | 728 (11.8) | 7406 (75.7) | < 0.001 |
| PaO_2_ (kPa) | 8.8 (2.9) | 8.5 (3.4) | 0.006 | 8.5 (3.1) | 8.6 (3.5) | 0.771 |
| PaCO_2_ (kPa) | 6.3 (2.1) | 6.4 (2.1) | 0.226 | 6.08 (1.8) | 6.6 (2.2) | <0.001 |
| pH | 7.39 (0.07) | 7.39 (0.07) | 0.188 | 7.4 (0.06) | 7.3 (0.07) | < 0.001 |
| Mild acidosis (pH 7.35-7.30) | 156 (6.9) | 1188 (8.6) | < 0.001 | 419 (6.8) | 919 (9.4) | < 0.001 |
| Severe acidosis (pH < 7.30) | 117 (5.2) | 991 (7.2) | < 0.001 | 259 (4.2) | 849 (8.7) | < 0.001 |
| Radiological findings:   - Normal or COPD-like - Bronchiectasis - Consolidation - Pleural effusion - Pneumothorax - Interstitial - Lung cancer | 1378 (61.4)  67 (3.0)  212 (9.4)  77 (3.4)  12 (0.5)  123 (5.5)  52 (2.3) | 7551 (54.8)  805 (5.8)  2757 (20.0)  378 (2.7)  21 (0.2)  673 (4.9)  347 (2.5) | < 0.001  < 0.001  < 0.001  0.051  0.001  0.169  0.708 | 3481 (56.4)  255 (4.1)  1020 (16.5)  177 (2.9)  15 (0.2)  314 (5.1)  158 (2.6) | 5427 (55.5)  614 (6.3)  1941 (19.9)  274 (2.8)  18 (0.2)  481 (4.9)  241 (2.5) | 0.242  < 0.001  < 0.001  0.456  0.364  0.425  0.441 |

Data expressed as mean (standard deviation) and absolute (relative) frequencies depending on the nature of the variable.

* p value calculated by Chi-squared test or Student T test for independent variables as appropriate.

FVC: forced vital capacity. FEV_1_: forced expiratory volume in the first second. PaO_2_: partial pressure of oxygen in arterial blood. PaCO_2_: partial pressure of carbon dioxide in arterial blood
