# Supplementary material for: Antibiotic Prescription for COPD Exacerbations Admitted to Hospital: European COPD Audit
Source: PLoS One. 2015 Apr 23;10(4):e0124374. doi: 10.1371/journal.pone.0124374 (PMC4408103; doi:10.1371/journal.pone.0124374)
Supplement: S2 Table — (DOCX) [file pone.0124374.s002.docx]

**S2 Table. Management-related variables between the study groups.**

|  | No antibiotic  (n=2245) | Antibiotic  (n=13773) | P value* | Not correct  (n=6176) | Correct  (n=9777) | P value* |
| --- | --- | --- | --- | --- | --- | --- |
| Respiratory ward | 1192 (53.2) | 7968 (57.9) | < 0.001 | 3337 (54.0) | 5795 (59.3) | < 0.001 |
| **Treatments before admission** | | | | | | |
| Short-acting bronchodilators (n) | 1478 (65.8) | 8993 (65.3) | 0.632 | 3956 (64.1) | 6474 (66.2) | 0.005 |
| Long-acting bronchodilators (n) | 1176 (52.4) | 7115 (51.7) | 0.539 | 3013 (48.8) | 5243 (53.6) | < 0.001 |
| Inhaled corticosteroids (n) | 1474 (65.7) | 9447 (68.8) | 0.003 | 4067 (65.9) | 6838 (69.9) | < 0.001 |
| Systemic corticosteroids (n) | 281 (30.7) | 1736 (33.4) | 0.110 | 1139 (18.4) | 1839 (18.8) | 0.573 |
| Antibiotics (n) | 318 (14.2) | 2884 (20.9) | < 0.001 | 1247 (20.2) | 1941 (19.9) | 0.612 |
| Methylxanthines (n) | 401 (17.9) | 2216 (16.1) | 0.036 | 918 (14.9) | 1697 (17.4) | < 0.001 |
| **Treatments during admission** | | | | | | |
| Short-acting bronchodilators (n) | 1924 (85.7) | 12670 (92.0) | < 0.001 | 5602 (90.7) | 8933 (91.4) | 0.153 |
| Inhaled corticosteroids (n) | 824 (36.7) | 4586 (33.3) | 0.002 | 1972 (31.9) | 3424 (35.0) | < 0.001 |
| Systemic corticosteroids (n) | 1583 (70.5) | 11604 (84.3) | < 0.001 | 5009 (81.1) | 8128 (83.1) | 0.001 |
| Methylxanthines (n) | 366 (16.3) | 1910 (13.9) | 0.003 | 773 (12.5) | 1499 (15.3) | < 0.001 |
| Diuretics (n) | 522 (23.3) | 3710 (26.9) | < 0.001 | 1523 (24.7) | 2694 (27.6) | < 0.001 |
| Oxygen (n) | 1644 (75.0) | 11958 (88.1) | < 0.001 | 5157 (84.8) | 8406 (87.1) | < 0.001 |
| Non-invasive MV (n) | 232 (10.7) | 1903 (14.3) | < 0.001 | 232 (3.9) | 1903 (20.1) | < 0.001 |
| Invasive MV (n) | 23 (1.1) | 263 (2.0) | 0.004 | 23 (0.4) | 263 (2.8) | < 0.001 |
| **Home treatments at discharge** | | | | | | |
| Short-acting bronchodilators (n) | 1479 (65.9) | 8514 (61.8) | < 0.001 | 3807 (61.6) | 6168 (63.1) | 0.067 |
| Long-acting bronchodilators (n) | 1521 (67.8) | 8878 (64.5) | 0.003 | 3975 (64.4) | 6385 (65.3) | 0.227 |
| Inhaled corticosteroids (n) | 1743 (77.6) | 11039 (80.1) | 0.006 | 4907 (79.5) | 7830 (80.1) | 0.331 |
| Systemic corticosteroids (n) | 1075 (47.9) | 7157 (52.0) | < 0.001 | 3250 (52.6) | 4961 (50.7) | 0.021 |
| Antibiotics (n) | 280 (12.5) | 6374 (46.3) | < 0.001 | 2640 (42.7) | 3980 (40.7) | 0.011 |
| Methylxanthines (n) | 507 (22.6) | 2672 (19.4) | 0.001 | 1170 (18.9) | 2002 (20.5) | 0.018 |
| Home oxygen (n) | 641 (29.4) | 4617 (34.6) | < 0.001 | 1800 (29.1) | 3439 (35.2) | < 0.001 |
| Home MV (n) | 125 (5.8) | 689 (5.2) | 0.232 | 148 (2.4) | 665 (6.8) | < 0.001 |

Data expressed as absolute (relative) frequencies. * p value calculated by Chi-squared test.
