# Supplementary material for: Antibiotic Prescription for COPD Exacerbations Admitted to Hospital: European COPD Audit
Source: PLoS One. 2015 Apr 23;10(4):e0124374. doi: 10.1371/journal.pone.0124374 (PMC4408103; doi:10.1371/journal.pone.0124374)
Supplement: S3 Table — (DOCX) [file pone.0124374.s003.docx]

**S3 Table. Guidelines compliance between the study groups.**

|  | No antibiot  (n=2245) | Antibiot  (n=13773) | P value* | Not correct  (n=6152) | Correct  (n=9801) | P value* |
| --- | --- | --- | --- | --- | --- | --- |
| Spirometry result available at admission | 1317 (58.7) | 8189 (59.5) | 0.487 | 3578 (57.9) | 5894 (60.3) | 0.003 |
| Arterial Blood Gas performed at admission | 1699 (78.1) | 11492 (85.1) | < 0.001 | 5042 (83.6) | 8111 (84.5) | 0.115 |
| Chest radiograph performed at admission | 2177 (97.0) | 13613 (98.8) | < 0.001 | 6076 (98.4) | 9651 (98.7) | 0.085 |
| Controlled oxygen therapy used | 1644 (75.0) | 11958 (88.1) | < 0.001 | 5157 (84.8) | 8406 (87.1) | < 0.001 |
| Short-acting bronchodilator use | 1924 (85.7) | 12670 (92.0) | <0.011 | 5602 (90.7) | 8933 (91.4) | 0.153 |
| Non-use of Intravenous methylxanthines | 1879 (83.7) | 11683 (86.1) | 0.003 | 5403 (87.5) | 8278 (84.7) | < 0.001 |
| Systemic corticosteroids given | 1583 (70.5) | 11604 (84.3) | < 0.001 | 5009 (81.1) | 8128 (83.1) | 0.001 |
| Antibiotic correctly given | 1344 (60.2) | 8457 (61.6) | 0.182 | – | – | – |
| NIV given if pH <7.35 and PaCO_2_>6kPa | 1419 (63.2) | 2637 (19.1) | < 0.011 | 4366 (88.9) | 6509 (82.9) | < 0.001 |
| IMV given if pH <7.25 and PaCO_2_>8kpa | 1569 (69.9) | 10608 (77.0) | < 0.001 | 4813 (77.9) | 7364 (75.3) | < 0.001 |
| Fulfilled all 10 recommendations | 251 (11.2) | 2193 (15.9) | < 0.001 | 5 (0.1) | 2439 (24.9) | < 0.001 |
