# Supplementary material for: Antibiotic Prescription for COPD Exacerbations Admitted to Hospital: European COPD Audit
Source: PLoS One. 2015 Apr 23;10(4):e0124374. doi: 10.1371/journal.pone.0124374 (PMC4408103; doi:10.1371/journal.pone.0124374)
Supplement: S1 Text — (DOC) [file pone.0124374.s004.doc]

**EUROPEAN COPD AUDIT**

**List of investigators**

**Steering Committee**

- C. Michael Roberts. Barts and The London School of Medicine and Dentistry Queen Mary University of London, London, UK
- Sylvia Hartl. Ludwig Boltzmann Institute of COPD and Respiratory Epidemiology, Vienna, Austria
- Jose Luis Lopez-Campos. Hospital Universitario Virgen del Rocío, Instituto de Biomedicina de Sevilla (IBiS), Sevilla. CIBER de Enfermedades Respiratorias (CIBERES). Instituto de Salud Carlos III, Madrid.

**Data analysis team**

- Francisco Pozo-Rodríguez. Hospital 12 de Octubre, Instituto de Investigación i+12, Madrid, Spain
- Jose Luis López-Campos. Hospital Universitario Virgen del Rocío, Instituto de Biomedicina de Sevilla (IBiS), Sevilla. CIBER de Enfermedades Respiratorias (CIBERES). Instituto de Salud Carlos III, Madrid.
- Ady Castro-Acosta. Hospital 12 de Octubre, Instituto de Investigación i+12, Madrid, Spain
- Víctor Abraira-Santos. Hospital Universitario Ramón y Cajal, IRYCIS, Madrid, Spain, Centre for Biomedical Research on Epidemiology and Public Health (CIBERESP). Instituto de Salud Carlos III. Madrid, Spain.
- Antonio López-Quilez. Department of Statistics and Operational Research. Universidad de Castellón, Castellón, Spain
- Juan Dorado. Consultoría estadística y de investigación clínica PERTICA

**National experts (by alphabetical order of the country)**

| Country | Name | Affiliation |
| --- | --- | --- |
| Austria | Otto Burghuber | Otto-Wagner Hospital, Vienna, Austria. |
|  | Robab Kohansal | Otto-Wagner Hospital, Vienna, Austria. |
| Belgium | Wim Janssens | Universitaire Ziekenhuizen, Leuven |
|  | Vincent Heinen | Centre Hospitalier Universitaire de Liège, Liège |
| Croatia | Neven Miculinic | Clinic for Respiratory Diseases, University Hospital “Zagreb” |
|  | Hrvoje Puretic | Clinic for Respiratory Diseases, University Hospital “Zagreb” |
| Greece | Nikos Tzanakis | University Hospital of Heraklion, Dept of Thoracic Medicine, Medical School, University of Crete |
|  | Epameinondas Nontas Kosmas | Respiratory Division PNOI  Metropolitan Hospital  Neo Faliro, Greece |
| Ireland | Suzanne McCormack | Irish thoracic society |
|  | Tim McDonell | St Vincent’s University Hospital, Dublin |
| Malta | Cynthia Farrugia -Jones | Mater Dei Hospital, Malta |
| Poland | Joanna Chorostowska-Wynimko | Institute of Tuberculosis and Lung Diseases in Warsaw |
| Romania | Florin Mihaltan | Institutul De Pneumoftiziologie "Marius Nasta", Bucharest |
|  | Miron Alexandru Bogdan | "Carol Davila" University of Medicine and Pharmacy, Bucharest. |
|  | Ioana Munteanu | Institutul De Pneumoftiziologie "Marius Nasta", Bucharest |
| Slovakia | Ivan Solovic | National Institute for TB, Lung Diseases and Thoracic Surgery |
|  | Ruzena Tkacova | University Hospital Kosice Pneumolog. Clinic |
| Spain | Francisco Pozo-Rodríguez | Hospital 12 de Octubre, Instituto de Investigación i+12, Madrid, Spain |
|  | Julio Ancochea | Hospital Universitario de La Princesa, Madrid |
| Switzerland | Daiana Stolz | Universitätsspital Basel, Basel |
| Turkey | Mehmet Polatli | Adnan Menderes Üniversitesi Tıp Fakültesi Göğüs Hastalıkları AD, Aydın |
|  | Elif Şen | Ankara Üniversitesi Tıp Fakültesi Göğüs Hastalıkları AD, Ankara |
| United Kingdom | Christine Bucknall | Stobhill General Hospital, Glasgow |
|  | Sally Welham | British Thoracic Society |
|  | Christopher Routh | British Thoracic Society |

**Project managers**

Moniek Haan. European Respiratory Society.

Marta Zarelli. European Respiratory Society.

Elmira Lechat.

Rhona J. Buckingham

**ERS COPD Audit Liaison Officer**

Guy Joos.

**Local investigators**

**AUSTRIA**

Dr Sascha Nassri, Landesklinikum Mostviertel Amstetten, Amstetten

Dr Nina Mitrovits, Krankenhaus der Barmherzigen Brüder Eisenstadt, Eisenstadt

Dr Otmar Schindler, Landeskrankenhaus Hörgas, Gratwein

Dr Christina Stöckl, Landeskrankenhaus Universitätsklinikum Graz, Graz

Dr Wolfgang Auer, Krankenhaus der Elisabethiner, Graz

Dr Johannes Bilek, Landesklinikum Thermenregion Hochegg, Grimmenstein

Dr Lorena Koch, Landeskrankenhaus Hohenems, Hohenems

Dr Thomas Jaritz, Landeskrankenhaus Klagenfurt, Klagenfurt

Dr Monika Kössler, Landesklinikum Krems, Krems/Donau

Dr Eva Kaufmann, Landeskrankenhaus Leoben Eisenerz, Leoben

Dr Michael Riedler, Allgemeines Krankenhaus der Stadt Linz, Linz

Dr. Elmar Brehm, Allgemeines Krankenhaus der Stadt Linz, Linz

Dr Sebastian Zillinger, Krankenhaus der Elisabethinen, Linz

Dr Franz Wimberger, Krankenhaus der Elisabethinen, Linz

Dr Daniel Krejci, Landeskrankenhaus Natters, Natters

Dr Jolande Schmid, Krankenhaus der Barmherzigen Schwestern, Ried/Innkreis

Mag. Gertraud Weiß, Landeskrankenhaus Salzburg, Salzburg

Dr. Lea Schirnhofer, Landeskrankenhaus Salzburg, Salzburg

Dr Bernd Lamprecht, Landeskrankenhaus Salzburg, Salzburg

Dr Gunther Schuller, Landeskrankenhaus Steyr, Steyr

Dr Heidrun Stetina-Zauner, Krankenhaus Vöcklabruck, Vöcklabruck

Dr Carolin Großruck, Klinikum Wels-Grieskirchen, Wels

Dr Bettina Heindl, Wilhelminenspital, 2. Med. Abt., Vienna

Dr David Dörfler, Krankenhaus Hietzing, Abt. für Atmungs- und Lungenerk., Vienna

Dr. Irene Werner, Krankenhaus Hietzing, Abt. für Atmungs- und Lungenerk., Vienna

Dr Anna Kropfmüller, Allgemeines Krankenhaus der Stadt Wien, Vienna

Dr Angelika Fichtenberg, Hartmannspital Wien, Vienna

Dr Elisabeth Vesely, Otto Wagner Spital, Vienna

Dr Alexander Feist, Otto Wagner Spital, Vienna

Dr Alexander Lindemeier, Kaiser Franz Josef Spital, 5. Med. Abt., Vienna

Dr Irene Firlinger, Otto Wagner Spital, 1. Interne Lungenabt., Vienna

Dr Leyla Ay, Rudolfstiftung, Vienna

Prof. Felix Stockenhuber, Landeskrankenhaus, Oberpullendorf

Dr WHR Kiss Heinrich, Landeskrankenhaus, Oberwart

Prim. Luschnig, Landeskrankenhaus, Bruck an der Mur

Dr Hannes Hoffmann, Landeskrankenhaus, Feldbach

Prof. Martin Mähring, Unfallkrankenhaus, Graz

Prof. Gerhard Schneider, Krankenhaus der Barmherzigen Brüder Graz-Eggenberg, Graz

Dr Wolfgang Thausing, Johannes von Gott-Pflegezentrum der Barmherzigen Brüder Graz-Kainbach, Graz-Ragnitz

Dr Erich Schaflinger, Krankenanstaltenverbund Mürzzuschlag-Mariazell, Mürzzuschlag

Dr. Heinz Stadler, Spitalsverbund Landeskrankenhaus Judenburg-Knittelfeld, Judenburg

Dr Heribert Walch, Landeskrankenhaus Graz-West, Graz

Dr Manfred Kuschnig, Krankenhaus der Elisabethinen, Klagenfurt

Dr Gerald Bruckmann, Krankenhaus, Spittal an der Drau

Dr Ralph Spernol, Landeskrankenhaus, Villach

Dr Albert Lingg, Landeskrankenhaus, Rankweil

Prof. Reinhard Haller, Krankenhaus Stiftung Maria-Ebene, Frastanz

Prof. Heinz Drexel, Landeskrankenhaus, Feldkirch-Tisis

Prof. Wolfgang Buchberger, Landeskrankenhaus Universitätsklinik, Innsbruck

Prof. Peter Lechleitner, Bezirkskrankenhaus, Lienz

Dr Josef Bazzanella, Bezirkskrankenhaus, Schwaz

Prof. Peter Sandbichler, Krankenhaus St Vinzenz, Zams

Prof. Klaus Gattringer, Bezirkskrankenhaus, Kufstein

Dr Werner Aufmesser, Krankenanstalt Radstadt Dr. Aufmesser, Radstadt

Dr Klaus Schwamberger, Aö Krankenhaus der Barmherzigen Brüder, Salzburg

Dr Gunther Ladurner, Christian Doppler-Klinik Salzburg - Universitätsklinikum der PMU, Salzburg

Prof. Reinhard Lenzhofer, Kardinal Schwarzenberg`sches Krankenhaus, Schwarzach im Pongau

Dr Werner Betzler, Allgemein öffentliches Krankenhaus Tamsweg des Landes Salzburg, Tamsweg

Dr Andreas Krauter, Krankenhaus der Barmherzigen Schwestern vom Hl. Vinzenz von Paul Linz, Linz

Dr Franz Thalhammer, Landeskrankenhaus, Bad Ischl

Dr Johann Schöppl, Krankenhaus St Josef Braunau, Braunau am Inn

Dr Johann Ecker, Landeskrankenhaus, Gmunden

Dr Florian Marberger, Landeskrankenhaus, Kirchdorf an der Krems

Dr Josef Macher, Diakonissen-Krankenhaus Linz, Linz

Dr Ekkehard Oberhammer, Landeskrankenhaus, Schärding

Dr Michael Berger, Öffentliche Sonderkrankenanstalt für Innere Medizin, Sierning

Dr Gustav Bartl, Krankenhaus der Barmherzigen Brüder Wien, Vienna

Dr Johannes Thoma, Krankenhaus der Barmherzigen Schwestern, Vienna

Prof. Klaus Klaushofer, Hanusch-Krankenhaus Wien, Vienna

Prof. Fellinger Erich, Sanatorium Hera, Vienna

Prof. Johannes Bonelli, Krankenhaus St Elisabeth, Vienna

Prof. Franz Böhmer, Sozialmedizinisches Zentrum Sophienspital der Stadt Wien, Vienna

Prof. Paul Bratusch-Marrain, Landesklinikum Waldviertel Horn-Allensteig, Horn

Dr Wolfgang Hintringer, Landesklinikum Weinviertel Korneuburg-Stockerau, Korneuburg

Dr Gerd Eichberger, Landesklinikum Donauregion Tulln, Tulln an der Donau

Dr Andreas Schneider, Landesklinikum St Pölten-Lilienfeld, St Pölten

Prof. Manfred Weissinger, Landesklinikum Waldviertel Zwettl-Gmünd-Waidhofen/Thaya, Zwettl

Dr Rupert Strasser, Landesklinikum Mostviertel Melk, Melk

Prof. Gerhard Lunglmayr, Landesklinikum Weinviertel Mistelbach, Mistelbach an der Zaya

Dr Johann Pidlich, Landesklinikum Thermenregion Baden-Mödling, Mödling

**BELGIUM**

Wim Janssens, Universitaire Ziekenhuizen, Leuven

Vincent Heinen, Centre Hospitalier Universitaire de Liège, Liège

Antoine Fremault, Grand Hôpital de Charleroi/Site St Joseph, Charleroi

Benoît Colinet, Grand Hôpital de Charleroi/Site St Joseph, Charleroi

Eric Derom, Universitair Ziekenhuis, Ghent

Marc Daenen, Ziekenhuis Oost-Limburg, Genk

Geert Tits, Sint-Andriesziekenhuis, Tielt

Valérie Van Damme, Sint-Andriesziekenhuis, Tielt

Vincent Ninane, CHU Saint-Pierre, Brussels

Giuseppe Liistro, Cliniques universitaires Saint-Luc, Brussels

Dominique Butenda, Centre Hospitalier du Bois de l'Abbaye et de Hesbaye, Seraing

Pierre Duchatelet, Réseau Hospitalier de Médecine Sociale, Baudour

Stéphane Kleis, Centre Hospitalier Peltzer - La Tourelle, Verviers

Michèle Ramaut, Centre Hospitalier Chrétien, Liège

Frédéric Fievet, Centre Hospitalier Chrétien, Liège

Jan Lamont, Algemeen Ziekenhuis Maria Middelares, Ghent

Ingel Demedts, Heilig-Hart Ziekenhuis, Roeselare/Menen

Kris Carron, Heilig-Hart Ziekenhuis, Roeselare/Menen

Philippe Bertrand, Heilig-Hart Ziekenhuis, Roeselare/Menen

Bart De Saedeleer, Algemeen Stedelijk Ziekenhuis, Geraardsbergen

Christian Quaden, Centre Hospitalier de Mouscron, Mouscron

Karine Laurent, Clinique Saint-Jean, Brussels

Rob Schildermans, Algemeen Ziekenhuis Sint-Lucas, Bruges

Philippe Rogiers, Algemeen Ziekenhuis Sint-Lucas, Bruges

Rudi Peché, CHU de Charleroi, Charleroi

Dominique Lauwers, CHU de Charleroi, Charleroi

Valérie Dufresne, CHU de Charleroi, Charleroi

Pierre Brancaleone, Centre Hospitalier Jolimont-Lobbes, La Louvière

Michel Vander Stappen, CHR Haute Senne, Soignies

Eric Marchand, Cliniques universitaires UCL de Mont-Godinne, Yvoir

Brigitte Janssens, AZ Sint-Lucas, Ghent

Peter Bogaerts, AZ Klina, Brasschaat

**CROATIA**

Dr Tatjana Tokić, General Hospital “Karlovac”

Dr Marijana Zadro-Bahnik, General Hospital “Karlovac”

Dr Tajana Jalušić-Glunčić, Special Hospital for Lung Diseases “Rockefellerova”

Dr Ljerka Glad, Special Hospital for Lung Diseases “Rockefellerova”

Prof. Kornelija Miše, Dept of Respiratory Medicine, University Hospital “Split”

Dr Jasminka Svalina-Grmuša, Dept of Respiratory Medicine, University Hospital “Split”

Dr Gordana Stjepanović, Dept of Pulmonology Petrinja, General Hospital “Sisak”

Dr Vesna Trkeš, Dept of Pulmonology Petrinja, General Hospital “Sisak”

Dr Suzana Sinovčić-Kolanović, General Hospital “Zadar”

Dr Željko Čulina, General Hospital “Zadar”

Dr Gordana Petrović, Dept of Pulmonology, Clinical Hospital “Osijek”

Dr Sanda Škrinjarić-Cincar, Dept of Pulmonology, Clinical Hospital “Osijek”

Dr Martina Hajduk, Hospital for Lung Diseases and Tuberculosis “Klenovnik”

Dr Ljubica Radmilović Hospital for Lung Diseases and Tuberculosis “Klenovnik”

Dr.Ljilajan Bulat-Kardum, Dept for Respiratory Medicine, University Hospital “Rijeka”

Dr Igor Barković, Dept for Respiratory Medicine, University Hospital “Rijeka”

Dr Miroslav Horvat, General Hospital “Čakovec”

Dr Ingrid Škvorc General Hospital “Čakovec”

Dr Neven Miculinić, Clinic for Respiratory Diseases, University Hospital “Zagreb”

Dr Hrvoje Puretić, Clinic for Respiratory Diseases, University Hospital “Zagreb”

**GREECE**

Nikos Tzanakis, University Hospital of Heraklion, Dept of Thoracic Medicine, Medical School, University of Crete

Giannis Giannarakis, University Hospital of Heraklion, Dept of Thoracic Medicine, Medical School, University of Crete

Giorgos Papadogiannis, University Hospital of Heraklion, Dept of Thoracic Medicine, Medical School, University of Crete

N.M. Siafakas, University Hospital of Heraklion, Dept of Thoracic Medicine, Medical School, University of Crete

Nikoletta Robina, Chest Diseases Hospital Sotiria–Athens, Dept of Respiratory Medicine, University of Athens

Petros Bakakos, Chest Diseases Hospital Sotiria–Athens, Dept of Respiratory Medicine, University of Athens

Sofianna Gennimata, Chest Diseases Hospital Sotiria–Athens, Dept of Respiratory Medicine, University of Athens

Tasos Palamidas, Chest Diseases Hospital Sotiria–Athens, Dept of Respiratory Medicine, University of Athens

Filippos Emmanouil, Chest Diseases Hospital Sotiria–Athens, Dept of Respiratory Medicine, University of Athens

Georgios Kaltsakas, Chest Diseases Hospital Sotiria–Athens, Dept of Respiratory Medicine, University of Athens

Manos Alchanatis, Chest Diseases Hospital Sotiria–Athens, Dept of Respiratory Medicine, University of Athens

Valantis Papageorgiou, Chest Diseases Hospital Sotiria–Athens, 1st Dept of Respiratory Medicine

Nikos Poulakis, Chest Diseases Hospital Sotiria–Athens, 1st Dept of Respiratory Medicine

Haris Lamprakis, Chest Diseases Hospital Sotiria–Athens, 2nd Dept of Respiratory Medicine

Fragiskos Vlastos, Chest Diseases Hospital Sotiria–Athens, 2nd Dept of Respiratory Medicine

Martha Andritsou, Chest Diseases Hospital Sotiria–Athens, 3rd Dept of Respiratory Medicine

Sylvia Dumitrou, Chest Diseases Hospital Sotiria–Athens, 3rd Dept of Respiratory Medicine

Nontas Kosmas, Chest Diseases Hospital Sotiria–Athens, 4th Dept of Respiratory Medicine

Antonis Bastas, Chest Diseases Hospital Sotiria–Athens, 4th Dept of Respiratory Medicine

Georgios Tsoukalas, Chest Diseases Hospital Sotiria–Athens, 4th Dept of Respiratory Medicine

George Hillas, Chest Diseases Hospital Sotiria–Athens, Dept of Respiratory and Critical Care Medicine

Kostas Sagris, Chest Diseases Hospital Sotiria–Athens, Dept of Respiratory and Critical Care Medicine

Demitris Veldekis, Chest Diseases Hospital Sotiria–Athens, Dept of Respiratory and Critical Care Medicine

Mixalis Toumbis, Chest Diseases Hospital Sotiria–Athens, 6th Dept of Respiratory Medicine

Eleftherios Zervas, Chest Diseases Hospital Sotiria–Athens, 7th Dept of Respiratory Medicine

Mina Gaga, Chest Diseases Hospital Sotiria–Athens, 7th Dept of Respiratory Medicine

Vasilis Kouranos, Chest Diseases Hospital Sotiria–Athens, 8th Dept of Respiratory Medicine

Nina Anagnostopoulou, Chest Diseases Hospital Sotiria–Athens, 8th Dept of Respiratory Medicine

Fotis Perlikos, Chest Diseases Hospital Sotiria–Athens, 12th Department of Respiratory Medicine

Nikos Vasilopoulos, Chest Diseases Hospital Sotiria–Athens, 12th Department of Respiratory Medicine

Mata Tsikrika, Sismanogleio General Hospital, Athens, 3rd Dept of Respiratory Medicine

Filia Diamantea, Sismanogleio General Hospital, Athens, 3rd Dept of Respiratory Medicine

Vlasis Polychronopoulos, Sismanogleio General Hospital, Athens, 3rd Dept of Respiratory Medicine

Olga Vartzioti, Sismanogleio General Hospital, Athens, 2nd Dept of Respiratory Medicine

Stelios Michailidis, Sismanogleio General Hospital, Athens, 2nd Dept of Respiratory Medicine

Isabella Srekensleger, Attikon General Hospital, 2nd Respiratory Medicine Dept, University of Athens Medical School

Stelios Loukidis, Attikon General Hospital, 2nd Respiratory Medicine Dept, University of Athens Medical School

Spyros Papiris, Attikon General Hospital, 2nd Respiratory Medicine Dept, University of Athens Medical School

Stavroula Kolokytha, Evagelismos Hospital, University of Athens Medical School, Dept of Pulmonary and Critical Care Medicine Ilias Siempos, Evagelismos Hospital, University of Athens Medical School, Dept of Pulmonary and Critical Care Medicine

Thoedoros Vasilakopoulos, Evagelismos Hospital, University of Athens Medical School, Dept of Pulmonary and Critical Care Medicine

Alexis Papadopoulos, Fleming General Hospital, Athens

Kostas Bartziokas, Fleming General Hospital, Athens

Katerina Haniotou, Fleming General Hospital, Athens

Eleni Karetsi, University Hospital of Larissa, Medical School, University Of Thessaly

Kostas Gourgoulianis, University Hospital of Larissa, Medical School, University Of Thessaly

Marios Froudarakis, University Hospital of Alexandroupolis, Dept of Respiratory Medicine, Thrace

Argyris Tzouvelekis, University Hospital of Alexandroupolis, Dept of Respiratory Medicine, Thrace

Paul Zaragoulidis, University Hospital of Alexandroupolis, Dept of Respiratory Medicine, Thrace

Demosthenes Bouros, University Hospital of Alexandroupolis, Dept of Respiratory Medicine, Thrace

Giorgos Crisofakis, Rethymno General Hospital, Rethymno, Crete, Dept of Respiratory Medicine

Kostas Kallergis, Rethymno General Hospital, Rethymno, Crete, Dept of Respiratory Medicine

Voula Mpousmpoukilia, General Hospital Of Kavala, Macedonia, Dept of Respiratory Medicine

Athanasia Pataka, Papanikoalaou General Hospital, University Respiratory Failure Unit, Aristotle University, Thessaloniki

Paraskevi Argyropouloy-Paraka, Papanikoalaou General Hospital, University Respiratory Failure Unit, Aristotle University, Thessaloniki

Dionisis Spyratos, G. Papanikoalaou General Hospital, Pulmonary Dept, General Hospital, Aristotle University of Thessaloniki, Thessaloniki

Kostas Zarogoulidis, G. Papanikoalaou General Hospital, Pulmonary Dept, General Hospital, Aristotle University of Thessaloniki, Thessaloniki

Maria Konoglou, G. Papanikoalaou General Hospital, 1st Dept of Respiratory Medicine, Thessaloniki

Eva Fouka, G. Papanikoalaou General Hospital, 1st Dept of Respiratory Medicine, Thessaloniki

Nikos Galanis, G. Papanikoalaou General Hospital, 2nd Dept of Respiratory Medicine, Thessaloniki

George Spyropoulos, G. Papanikoalaou General Hospital, 2nd Dept of Respiratory Medicine, Thessaloniki

Venetia Tsara, G. Papanikoalaou General Hospital, 2nd Dept of Respiratory Medicine, Thessaloniki

Kyriakos Karkoylis, University Hospital of Patras, Patra, Dept of Respiratory Medicine

Kostas Spiropoulos, University Hospital of Patras, Patra, Dept of Respiratory Medicine

Thanasis Konstantinidis, University Hospital of Ioaninna, Ioannina, Dept of Respiratory Medicine

Stavros Konstantopoulos, University Hospital of Ioaninna, Ioannina, Dept of Respiratory Medicine

Anna Gavriilidou, Papageorgiou General Hospital, Thessaloniki, Dept of Respiratory Medicine

Mariana Kakoura, Papageorgiou General Hospital, Thessaloniki, Dept of Respiratory Medicine

Elefteria Hainis, Corfu General Hospital, Corfu, Dept of Respiratory Medicine

Kyriakos Hainis, Corfu General Hospital, Corfu, Dept of Respiratory Medicine

Stavroula Amanetopoulou, General Hospital of Nikaia, Athens, Dept of Respiratory Medicine

Georgios Mathioudakis, General Hospital of Nikaia, Athens, Dept of Respiratory Medicine

Petros Oikonomidis, Filiates General Hospital, Thesprotia, Dept of Respiratory Medicine

Kostas Papas, Venizeleio General Hospital Heraklion, Crete, Dept of Respiratory Medicine

Nikos Mpachlitzanakis, Hania General Hospital, Crete, Dept of Respiratory Medicine

Vikki Krietsepi, Hania General Hospital, Crete, Dept of Respiratory Medicine

Manolis Daoukakis, 401 General Army Hospital , Athens, Dept of Pneumonology

Kostas Psathakis, 401 General Army Hospital , Athens, Dept of Pneumonology

Kostas Tsintiris, 401 General Army Hospital , Athens, Dept of Pneumonology

Evi Argiana, Asklipieion Hospital Heraklion, Crete

Tina Lamprini, General Hospital Of Sparta, Lakonia, Peloponissos

**IRELAND**

Dr Michael Henry, Cork University Hospital

Ms Bernadette Bowen, Cork University Hospital

Dr Edward Mc Swiney, Cork University Hospital

Dr Liam Cormican, Connolly Hospital Dublin

Dr Kenneth Bolger, Connolly Hospital Dublin

Dr Mazan Al Alawi, Connolly Hospital Dublin

Dr Sophie Kerr, Connolly Hospital Dublin

Ms Michele Cuddihy, Connolly Hospital Dublin

Ms Pamela Quinn, Connolly Hospital Dublin

Dr Joan Power, Naas General Hospital

Dr Caroline O Connell, Naas General Hospital

Dr Ijad Kamal, Naas General Hospital

Dr Shabhaz Sheikh, AMNCH, Tallaght, Dublin

Dr Eddie Moloney, AMNCH, Tallaght, Dublin

Dr Terry O’Connor, Mercy University Hospital, Cork

Ms Bernie O’Connor, Mercy University Hospital, Cork

Prof. Tim McDonnell, St Vincent’s University Hospital, Dublin

Dr Patrick Mitchell, St Vincent’s University Hospital, Dublin

Ms Mary Frances O’Driscoll, St Michael’s Hospital, Dublin

Dr Silke Ryan, St Michael’s Hospital, Dublin

Dr Vera Keatings, Letterkenny General Hospital

Mrs Sonya Murray, Letterkenny General Hospital

Dr Navid Valizadeh, Letterkenny General Hospital

Dr Liam Doherty, Bon Secours Hospital, Cork

Dr Notradamus Medina, Bon Secours Hospital, Cork

Dr Rory O’Donnell, St James’s Hospital, Dublin

Dr Parthiban Nadarajan, St James’s Hospital, Dublin

Ms Bettina Korn, St James’s Hospital, Dublin

Mr Stephen Shelly, St James’s Hospital, Dublin

Dr Seamus Linnane, Blackrock Clinic, Dublin

**MALTA**

Dr Cynthia Farrugia Jones

Dr Eleonor Gerada

Dr Josephine Bigeni

**POLAND**

Paweł Kuca, Instytut Gruźlicy i Chorób Płuc w Warszawie

Monika Targowska, Instytut Gruźlicy i Chorób Płuc w Warszawie

Małgorzata Czajkowska-Malinowska, Kujawsko-Pomorskie Centrum Pulmonologii w Bydgoszczy

Piotr Dąbrowiecki, CSK MON Wojskowy Instytut Medyczny w Warszawie

Andrzej Chciałowski, CSK MON Wojskowy Instytut Medyczny w Warszawie

Beata Mokwa, Szpital Specjalistyczny im. J. K. Łukowicza w Chojnicach

Małgorzata Kaczmarek, Szpital Specjalistyczny im. J. K. Łukowicza w Chojnicach

Joanna Chamera-Janicka, SPZOZ w Proszowicach

Edyta Kowalska, SPZOZ w Proszowicach

Wojciech Skucha, SPZOZ w Proszowicach

Mariola Gacek, Specjalistyczny Zespół Chorób Płuc i Gruźlicy w Bystrej

Małgorzata Konior, Specjalistyczny Zespół Chorób Płuc i Gruźlicy w Bystrej

Agnieszka Sadzikowska, Szpital Powiatowy w Chrzanowie

Jolanta Serafin-Bromblik, Szpital Powiatowy w Chrzanowie

Karolina Adamczyk-Bąk, Uniwersyteckie Centrum Kliniczne, Gdański Uniwersytet Medyczny

Karolina Kita, Uniwersyteckie Centrum Kliniczne, Gdański Uniwersytet Medyczny

Joanna Apiyo, Regionalny Szpital Specjalistyczny im dr. Władysława Biegańskiego.w Grudziądzu

Lechosław Jaszczołt, SP ZOZ Szpital Wojewódzki w Jeleniej Górre

Bożena Ziółkowska-Graca, Szpital Uniwersytecki w Krakowie

Aleksander Kania, Szpital Uniwersytecki w Krakowie

Dorota Daniel, Krakowski Szpital Specjalistyczny im. Jana Pawła II

Renata Bunk, Krakowski Szpital Specjalistyczny im. Jana Pawła II

Sebastian Majewski, Uniwersytecki Szpital Kliniczny nr 1 w Łódzi

Maciej Ciebiada, Uniwersytecki Szpital Kliniczny nr 1 w Łódzi

Magdalena Kostrzewska, Szpital Kliniczny Przemienienia Pańskiego Uniwersytetu Medycznego w Poznaniu

Mariusz Torous, Szpital Kliniczny Przemienienia Pańskiego Uniwersytetu Medycznego w Poznaniu

Marek Kamiński, Specjalistyczny Szpital im. prof. Alfreda Sokołowskiego w Szczecinie

Renata Bonikowska, Specjalistyczny Szpital im. prof. Alfreda Sokołowskiego w Szczecinie

Joanna Neuman, Specjalistyczny Szpital im. prof. Alfreda Sokołowskiego w Szczecinie

Beata Chmielowicz, Akademicki Szpital Kliniczny we Wrocławiu

Robert Pawłowicz, Akademicki Szpital Kliniczny we Wrocławiu

Aneta Kowal, Dolnośląskie Centrum Gruźlicy i Chorób Płuc

Monika Kosacka, Dolnośląskie Centrum Gruźlicy i Chorób Płuc

Anna Gostkowska-Malec, Dolnośląskie Centrum Gruźlicy i Chorób Płuc

Małgorzata Maksymiak, Samodzielny Publiczny Szpital Kliniczny nr 3 w Zabrzu

Justyna Wyrwoł, Samodzielny Publiczny Szpital Kliniczny nr 3 w Zabrzu

Roksana Małota, Samodzielny Publiczny Szpital Kliniczny nr 3 w Zabrzu

Wanda Lutogniewska, Samodzielny Publiczny Szpital Kliniczny nr 3 w Zabrzu

Jarosław Sokołowski, Szpital Wojewódzki we Włocławku

Jolanta Helińska, Szpital Wojewódzki we Włocławku

Paweł Wudarski, Wojewódzki Szpital Zespolony im. L. Rydygiera w Toruniu

Ewa Trawińska, Wojewódzki Szpital Zespolony im. L. Rydygiera w Toruniu

Tadeusz Zielonka, Szpital Czerniakowski w Warszawie

Jan Lesiński, Szpital Czerniakowski w Warszawie

Zygmunt Konieczny, Samodzielny Publiczny Zakład Opieki Zdrowotnej ZOZ w Głuchołazach

Anna Piskorowska-Pliś, Szpital Wojewódzki w Opolu

**ROMANIA**

Traian Mihăescu, Spital Clinic Pneumologie, Iasi

Cojocaru Cristian, Spital Clinic Pneumologie, Iasi

Monica Carmen Pop, Spitalul Clinic De Pneumoftiziologie "Leon Daniello" Cluj-Napoca, Cluj-Napoca

Mihaela Pop, Spitalul Clinic De Pneumoftiziologie "Leon Daniello" Cluj-Napoca, Cluj-Napoca

Gabriela Jimborean, Spitalul Clinic Judeţean Mureş, Mures

Corina Budin, Spitalul Clinic Judeţean Mureş, Mures

Voicu Tudorache, Spitalul Clinic De Boli Infecţioase Şi Pneumoftiziologie "Dr. Victor Babes", Timisoara

Zeno-Ioan Frăţila, Spitalul Clinic De Boli Infecţioase Şi Pneumoftiziologie "Dr. Victor Babes", Timisoara

Mimi Floarea Nițu, Spitalul Clinic De Boli Infecţioase Şi Pneumoftiziologie „Dr. Victor Babeş“, Craiova

Mihai Olteanu, Spitalul Clinic De Boli Infecţioase Şi Pneumoftiziologie „Dr. Victor Babeş“, Craiova

Cristina Oana Arghir, Spitalul Clinic De Pneumoftiziologie, Constanta

Mihaela Trenchea, Spitalul Clinic De Pneumoftiziologie, Constanta

Ovidiu Frâncu, Spitalul De Pneumoftiziologie, Sibiu

Elena Maria Scridon, Spitalul De Pneumoftiziologie, Sibiu

Gheorghe Nini, Spitalul TBC Arad, Arad

Ioan Stelian Morariu, Spitalul TBC Arad, Arad

Sorina Oana Alexandrescu, Spitalul De Pneumoftiziologie Brasov, Brasov

Mureşan Alina, Spitalul De Pneumoftiziologie Brasov, Brasov

Florin Mihălțan, Institutul De Pneumoftiziologie "Marius Nasta", Bucharest

Miron Bogdan, Institutul De Pneumoftiziologie "Marius Nasta", Bucharest

Alina Croitoru, Institutul De Pneumoftiziologie "Marius Nasta", Bucharest

Liliana Grigoriu, Institutul De Pneumoftiziologie "Marius Nasta", Bucharest

Ioana Munteanu, Institutul De Pneumoftiziologie "Marius Nasta", Bucharest

**SLOVAKIA**

Ivan Solovic, National Institute for TB, Lung Diseases and Thoracic Surgery

Ruzena Tkacova, University Hospital Kosice Pneumolog. Clinic

Ivan Skyba, University Hospital Kosice Pneumolog. Clinic

Ivan Kocan, University Hospital Martin Pneumolog. Clinic

**SPAIN**

José Calvo Bonachera, Complejo Hospitalario Torrecárdenas

Maria Paz Martínez Cortes, Complejo Hospitalario Torrecárdenas

Bernardino Alcázar Navarrete, Complejo Hospitalario de Jaén

Armando Falces Sierra, Hospital de La Línea de la Concepción

Marisol Arenas de la Riva, Hospital Universitario Reina Sofía

Rosa Vázquez Oliva, Hospital Infanta Elena

Fernando Hernández Utrera, Hospital Infanta Elena

Juan Manuel Bravo Santervás, E.P.H.A.G. Alto Guadalquivir (Andújar)

Francisco Canales Cid, E.P.H.A.G. Alto Guadalquivir (Andújar)

Jose Luis López Campos, Hospital Universitario Virgen del Rocío

Pablo Pérez Navarro, Hospital Universitario Virgen del Rocío

Inmaculada Alfageme Michavila, Hospital Universitario Valme

Zulema Palacios Hidalgo, Hospital Universitario Valme

Fernando Romero, Hospital Puerta del Mar

Isidro Blanco, Hospital Puerta del Mar

Gregorio Soto Campos, Hospital General de Jeréz de la Frontera

Aida García Cuesta, Hospital General de Jeréz de la Frontera

Carlos Rueda, Hospital Comarcal de Vélez Málaga

Alicia Conde, Hospital Universitario San Cecilio

Joaquín Carlos Costan Galicia, Hospital Clínico Universitario Lozano Blesa

Laura Anoro Abenoza, Hospital Clínico Universitario Lozano Blesa

Salvador Bello Dronda, Hospital Miguel Servet

Andrés Sánchez Barón, Hospital Miguel Servet

Luis Borderias, Hospital San Jorge de Huesca

Helena Briz Muñoz, Hospital San Jorge de Huesca

Cristina Martínez, I.N. Silicosis de Asturias

Marta García Clemente, I.N. Silicosis de Asturias

Ana Pando Sandoval, I.N. Silicosis de Asturias

Francisco Julián López González, I.N. Silicosis de Asturias

Aida Quero Martínez, I.N. Silicosis de Asturias

Fernando Álvarez Navascues, Hospital San Agustín de Avilés

Manuel Villanueva Montes, Hospital San Agustín de Avilés

Teresa Pascual Pascual, Hospital de Cabueñes Gijón

Antonio Cascales García, Hospital Can Misses, Ibiza

Álvaro de Astorza, Hospital Can Misses, Ibiza

Salvador Pons, Hospital Son Llàtzer

Maria Rosa Irigaray, Hospital de Manacor

Maria José Cons, Hospital de Manacor

Borja García-Cosío Piqueras, Hospital Son Dureta

Rocío Córdova Díaz, Hospital Son Dureta

Magdalena Alonso, Hospital Nuestra Señora de la Candelaria, Tenerife

Ruth Pitti, Hospital Nuestra Señora de la Candelaria, Tenerife

Luisa Eiroa González, Hospital Nuestra Señora de la Candelaria, Tenerife

Ana Velázquez, Hospital Nuestra Señora de la Candelaria, Tenerife

Ramón Agüero Balbín, Hospital Marqués de Valdecilla

Carlos Amado Diago, Hospital Marqués de Valdecilla

Beatriz Abascal Bolado, Hospital Marqués de Valdecilla

Miguel Zabaleta Murguiondo, H de Laredo

Mar García Pérez, Hospital de Sierrallana

Néstor Soler Porcar, Hospital Clinic Barcelona

Silvia Valls, Hospital Clinic Barcelona

Nuria Rodríguez Lázaro , Hospital Comarcal de L´alt Penedés, Vilefrance de Penedés

Joaquín Gea Giral, Hospital del Mar

Roser Pedreny, Hospital del Mar

Sergi Pascual, Hospital del Mar

Ignasi Garcia Olivé, Hospital Universitari Germans Trias i Pujol

Carlos Martínez , Hospital Universitari Germans Trias i Pujol

Ramona Hervas, Hospital Universitari Germans Trias i Pujol

Esther Rodríguez González, Hospital Vall d´Hebron

Eva Tapia Melechon, Hospital Vall d´Hebron

Ángeles Barrio Guirado, Hospital Vall d´Hebron

Milagros Gándara Sanz, Hospital Vall d´Hebron

David Lobillo Lopez, Hospital Vall d´Hebron

Eugenia Bueno Portela, Hospital Vall d´Hebron

Luis Lores Obradors, Hospital General Par Sanitari Sant Joan de Déu

Eduard Monso, Hospital Parc Taulí de Sabadell

Laia Seto Gort, Hospital Parc Taulí de Sabadell

Leonardo Esteban, Hospital Joan XXIII de Tarragona

Manel Haro Estarriol, Hospital Doctor Josep Trueta de Girona

Mª José Peirón Puyal, Hospital Virgen de la Luz (Cuenca)

José Maria Peñas Herrero, Hospital Virgen de la Luz (Cuenca)

Maria Eugenia Casado López, Hospital Virgen de la Luz (Cuenca)

Rosario Vargas Gonzalez, Hospital Virgen de la Luz (Cuenca)

Encarnación López Gabaldón, Hospital Virgen de la Salud (Toledo)

Javier Quiles La Puerta, Hospital Virgen de la Salud (Toledo)

Raúl Hidalgo Carvajal, Hospital Virgen de la Salud (Toledo)

Galo Fernández Zapata, Hospital Virgen de la Salud (Toledo)

Isabel García San José, Hospital Virgen de la Salud (Toledo)

Yamilex Urbano Aranda, Hospital Virgen de la Salud (Toledo)

Beatriz Cadavid Rodríguez, Hospital Virgen de la Salud (Toledo)

Jesus Fernández Frances, Hospital Universitario de Guadalajara

Elisabeth Guzmán Robles, Hospital Universitario de Guadalajara

Juan Pablo Rodríguez Gallego, Hospital Universitario de Guadalajara

José Celdrán Gil, Hospital Nuestra Señora del Prado (Talavera)

Jesús Reyes Hernández, Hospital Nuestra Señora de Sonsoles

José Eugenio Alonso Muñoz, Hospital Nuestra Señora de Sonsoles

Graciliano Estrada Trigueras, Hospital General de Segovia

José Luis Orcastegui Candial, Complejo Asistencial de Soria, Hospital Santa Bárbara

Isabel Ramos Cancelo, Complejo Asistencial de Soria, Hospital Santa Bárbara

Ruth García García, Complejo Asistencial de Soria, Hospital Santa Bárbara

Carlos Disdier, Hospital Universitario de Valladolid

Enrique Macías, Hospital Universitario de Valladolid

Jaime Sanabria, Hospital Universitario de Valladolid

Angela Peñaloza, Hospital Universitario de Valladolid

Félix Del Campo Matías , Hospital Rio Hortega

Juan Ortíz de Saracho, Hospital del Bierzo

Pedro Cancelo, Hospital Santos Reyes Aranda de Duero

Maria Ángeles Fernández Jorge, Complejo Asistencial de Palencia

Maria Inés Carrascosa, Hospital de Santiago

Silvia Dorronsoro, Hospital de Zumarraga

Laura Tomás, Hospital de Txagorritxu

Pilar Marín, Hospital de Cruces

José María Antoñana, Hospital de Cruces

Mikel Egurrola, Hospital de Galdakao

Cristóbal Esteban, Hospital de Galdakao

Jesús Camino, Hospital de San Eloy

Susana Chic Palacin, Hospital de Mendaro

José Ignacio Royo, Hospital de Mendaro

Begoñe Salinas, Hospital de Basurto

Igor Iturbe, Hospital de Basurto

Mikel Temprano, Hospital de Mondragón

Juan Antonio Miguel Arce, Hospital de Bidasoa

José Antonio Gutiérrez Lara, Hospital Infanta Cristina (Badajoz)

José Antonio Marín Torrado, Hospital Infanta Cristina (Badajoz)

Estefania Molina Ortiz, Hospital Infanta Cristina (Badajoz)

Lourdes Cañón Barroso, Hospital Infanta Cristina (Badajoz)

Juan Antonio Riesco Miranda, Hospital San Pedro de Alcántara

Elena Badarán, Hospital San Pedro de Alcántara

Maria José López Jiménez, Hospital San Pedro de Alcántara

Alfonso García Guisado, Hospital San Pedro de Alcántara

Mirian Torres Gonzalez, Hospital San Pedro de Alcántara

Germán García de Vinuesa, Mérida

Marisa Dolores Corbacho, Hospital Povisa

Jesús Gonzalez Ayude, Hospital Povisa

Alberto Fernández Villar, Complejo Hospitalario Universitario de Vigo

Cristina Represas Represas, Complejo Hospitalario Universitario de Vigo

Maria Isabel Botana, Complejo Hospitalario Universitario de Vigo

Pedro Marcos Velázquez, Complejo Hospitalario de Ourense

Isaura Parente Lamelas, Complejo Hospitalario de Ourense

Mariluz Santalla Martínez, Complejo Hospitalario de Ourense

Manuel M Barrón Medrano, Hospital San Pedro de la Rioja

Carlos Ruíz Martínez, Hospital San Pedro de la Rioja

Maria del Carmen Mascareño, Hospital San Pedro de la Rioja

Francisco Campano, Hospital San Pedro de la Rioja

Carlos Álvarez, Hospital Universitario 12 de Octubre

Virginia Pérez González, Hospital Universitario 12 de Octubre

Gema Rodríguez Trigo, Hospital Universitario Clínico San Carlos

Enrique Zamora García, Hospital de la Princesa

María del Valle Somiedo, Hospital de la Princesa

Sara Yamamoto, Hospital de la Princesa

Jose Andrés García Romero de Tejada, Hospital de la Princesa

Gonzalo Segrelles Calvo, Hospital de la Princesa

Rosa Mar Gómez Púnter, Hospital de la Princesa

Antolin López Viña, Hospital Puerta de Hierro

Miriam Aguilar, Hospital Puerta de Hierro

Rosa Malo de Molina, Hospital Puerta de Hierro

Patricia Minguez Clemente, Hospital Puerta de Hierro

Andrea Trisán Alonso, Hospital Puerta de Hierro

Manuel Valle Falcones, Hospital Puerta de Hierro

Sergio Salgado Aranda, Hospital Sureste Arganda del Rey

Mónica Gómez García, Hospital Sureste Arganda del Rey

María Piñeiro Martínez, Hospital Sureste Arganda del Rey

German Peces Barba, Fundación Jiménez Díaz

Sandra Pelícano, Fundación Jiménez Díaz

José Fernández, Fundación Jiménez Díaz

Javier Jareño, Hospital Central de la Defensa (Gómez Ulla)

Sergio Campos Tellez, Hospital Central de la Defensa (Gómez Ulla)

Raúl Moreno Zabaleta, Hospital Infanta Sofía (SS Reyes)

María Teresa Ramírez Prieto, Hospital Infanta Sofía (SS Reyes)

Maria Antonia Juretschke Moragues, Hospital de Getafe

Pilar Andres, Hospital de Getafe

David Lin, Hospital de Getafe

Francisco García Río, Hospital Universitario La Paz

Mari Angeles Ruiz-Cobos, Hospital del Henares

Belén Arnalich Jimenez, Hospital del Henares

Álvaro Casanova Espinosa, Hospital del Henares

Eva de Santiago Delgado, Hospital del Henares

José Miguel Rodríguez, Hospital Gregorio Marañón

Jorge Eisner Garcia, Hospital Gregorio Marañón

Salvador Diaz Lobato, Hospital Ramón y Cajal

Esteban Pérez Rodríguez, Hospital Ramón y Cajal

Beatriz Jara Chinarro, Hospital Infanta Cristina

Maria Jesús Buendia, Hospital Infanta Leonor

África Alcorta Mesas, Hospital Infanta Leonor

Carmen Matesanz Ruiz, Hospital Infanta Leonor

Vanesa Lores Gutiérrez, Hospital Infanta Leonor

María Belén López-Muñiz Ballesteros, Hospital Infanta Leonor

Julio Hernández Vázquez, Hospital Infanta Leonor

Yunelsey Anta Mejías, Hospital Infanta Leonor

Soledad Alonso Viteri, Hospital Príncipe de Asturias Alcalá Henares

Alicia Ferreira, Hospital Príncipe de Asturias Alcalá Henares

Antonio Ruiz, Hospital Príncipe de Asturias Alcalá Henares

Concepción Losada, Hospital Príncipe de Asturias Alcalá Henares

Esther Alonso Peces, Hospital Príncipe de Asturias Alcalá Henares

Gerardo Vázquez, Hospital Príncipe de Asturias Alcalá Henares

Julio Flores, Hospital Príncipe de Asturias Alcalá Henares

Dolores Álvaro, Hospital de Móstoles

Natividad Quílez Ruíz-Rico, Hospital de Móstoles

Raquel Pérez Rojo, Hospital de Móstoles

María Vázquez Mezquita, Hospital de Móstoles

Olga Navarrete, Hospital de Móstoles

Silvia Sánchez, Hospital de Móstoles

Asunción Perpina, Hospital Severo Ochoa (Leganés)

Pilar Alba, Hospital Severo Ochoa (Leganés)

Damián Malia Alvarado, Hospital de los Arcos

Nuria Castejón Pina, Hospital de los Arcos

Jose Antonio Ros Lucas, Hospital de los Arcos

Ada Luz Andreu Rodríguez, Hospital de los Arcos

Juan Miguel Sánchez Nieto, Hospital General Universitario Morales Meseguer

Roberto Bernabeu Mora, Hospital General Universitario Morales Meseguer

Manuel Castilla Martínez, Hospital General Universitario Morales Meseguer

Olga Meca Birlanga, Hospital General Universitario Morales Meseguer

Pilar Berlinches, Hospital Santa Lucía

Inés Bernal, Hospital Santa Lucía

Javier Hueto Pérez de Heredia, Complejo Hospitalario de Navarra

Joan Boldu Mitgans, Complejo Hospitalario de Navarra

Pilar Cebollero Rivas, Complejo Hospitalario de Navarra

José Antonio Cascante Rodrígo, Complejo Hospitalario de Navarra

Víctor Manuel Eguía Astibia, Complejo Hospitalario de Navarra

Idoya Pascal Martínez, Complejo Hospitalario de Navarra

Pablo Catalán Serra, Hospital de Requena

Eva Martínez Moragon, Hospital de Sagunto

Jose Manuel Querol, Hospital de Orihuela

Concha Pellicer, Hospital Frances de Borja, Gandia

Eusebi Chiner Vives, Hospital San Joan de Alicante

Cristina Senent Español, Hospital San Joan de Alicante

José Norberto Sancho Chust, Hospital San Joan de Alicante

Ángela Cervera Juan, Hospital Dr. Peset

Estrella Fernández-Fabrellas, Hospital Dr. Peset

Anna Santabasilisa, Hospital Dr. Peset

Susana Herrera, Hospital Dr. Peset

Ruben Lera, Hospital Dr. Peset

Cristina Miralles, Hospital Dr. Peset

Belen Orosa, Hospital Dr. Peset

Elsa Naval Sendra, Hospital de la Ribera

Inmaculada Lluch Tortajada, Hospital de la Ribera

Maria Cruz González, Hospital Clínico de Valencia

Paola Lisseth Ordoñez Gómez, Hospital Clínico de Valencia

Erick Leonardo Monclou Garzón, Hospital Clínico de Valencia

Maria Dolores Martínez Pitarch, Hospital Clínico de Valencia

Lucia Gil Maneu, Hospital Clínico de Valencia

Margarita Marín Royo, Hospital General de Castellón

German Llavador, Hospital General de Castellón

Alfonso Martinez, Hospital General de Castellón

Juliana Rissi, Hospital General de Castellón

Maria Jose Bueso, Hospital General de Castellón

**SWITZERLAND**

Dr Jürg Barandun, Lungenzentrum Hirslanden, Zürich

Dr Heinz Borer, Bürgerspital Solothurn, Solothurn

Dr Albrecht Breitenbücher, Kantonsspital Bruderholz, Bruderholz

Dr Kathleen Jahn, Kantonsspital Bruderholz, Bruderholz

Prof. Martin Brutsche, Kantonsspital St Gallen, St Gallen

Dr Jochen Rüdiger, Kantonsspital St Gallen, St Gallen

Dr René Fiechter, GZO Spital Wetzikon, Wetzikon

Prof. Thomas Geiser, Inselspital, Berne

Dr Michael Grob, Spitalzentrum Biel, Biel

Dr Erich Helfenstein, Lungenpraxis Hirslanden – Klinik St Anna, Lucerne

Dr Lilian Junker, Spital Thun, Thun

Dr Werner Karrer, Luzerner Höhenklinik, Crans-Montana

Dr Patrick Brun, Luzerner Höhenklinik, Crans-Montana

Dr Erich Köhler, Kantonsspital Liestal, Liestal

Dr Eva Koltai, Spital Laufenburg, Laufenburg

Dr Marc Maurer, Kantonsspital Aarau, Aarau

Dr Daniel Schilter, Spital Bern-Tiefenau, Berne

Dr Tino Schneider, Kantonsspital Chur, Chur

Dr Thomas Sigrist, Zuger Kantonsspital AG, Baar

Prof. Markus Solèr, St Claraspital, Basel

Prof. Robert Thurnheer, Thurgauer Kantonsspital, Münsterlingen

Prof. Daiana Stolz, Universitätsspital Basel, Basel

**TURKEY**

Dr Mehmet Polatlı, Adnan Menderes Üniversitesi Tıp Fakültesi Göğüs Hastalıkları AD, Aydın

Dr Aykut Çilli, Akdeniz Üniversitesi Tıp Fakültesi Göğüs Hastalıkları AD, Antalya

Dr Fulya Danacı, Akdeniz Üniversitesi Tıp Fakültesi Göğüs Hastalıkları AD, Antalya

Dr Nurhan Sarıoğlu, Akyazı Devlet Hastanesi, Sakarya

Dr Elif Şen, Ankara Üniversitesi Tıp Fakültesi Göğüs Hastalıkları AD, Ankara

Dr Leyla Sağlam, Atatürk Üniversitesi Tıp Fakültesi Göğüs Hastalıkları AD, Erzurum

Dr Elif Yılmazel Uçar, Atatürk Üniversitesi Tıp Fakültesi Göğüs Hastalıkları AD, Erzurum

Dr Esra Kunt Uzaslan, Bursa Uludağ Üniversitesi Tıp Fakültesi, Göğüs Hastalıkları AD, Bursa

Dr Aslı Görek Dilektaşlı, Bursa Uludağ Üniversitesi Tıp Fakültesi, Göğüs Hastalıkları AD, Bursa

Dr Ayşın Şakar Coşkun, Celal Bayar Üniversitesi Tıp Fakültesi Göğüs Hastalıkları AD, Manisa

Dr Uğur Gönlügür, Çanakkale Onsekiz Mart Üniversitesi Tıp Fakültesi Göğüs Hastalıkları AD, Çanakkale

Dr İsmail Hanta, Çukurova Üniversitesi Tıp Fakültesi Göğüs Hastalıkları AD,Adana

Dr Abdurrahman Şenyiğit, Dicle Üniversitesi Tıp Fakültesi Göğüs Hastalıkları AD, Diyarbakır

Dr Çetin Tanrıkulu, Dicle Üniversitesi Tıp Fakültesi Göğüs Hastalıkları AD, Diyarbakır

Dr Cengizhan Sezgi, Dicle Üniversitesi Tıp Fakültesi Göğüs Hastalıkları AD, Diyarbakır

Dr Abdurrahman Abakay, Dicle Üniversitesi Tıp Fakültesi Göğüs Hastalıkları AD, Diyarbakır

Dr. Abdullah Sayıner, Ege Üniversitesi Tıp Fakültesi Göğüs Hastalıkları AD, İzmir

Dr Alev Gürgün, Ege Üniversitesi Tıp Fakültesi Göğüs Hastalıkları AD, İzmir

Dr Gamze Kırkıl, Fırat Üniversitesi Tıp Fakültesi Göğüs Hastalıkları AD, Elazığ

Dr Nurdan Köktürk, Gazi Üniversitesi Tıp Fakültesi Göğüs Hastalıkları AD, Ankara

Dr Arzu Balkan, Gülhane Askeri Tıp Akademisi (GATA) Hastanesi, Göğüs Hastalıkları AD, Ankara

Dr Esen Kıyan, İstanbul Üniversitesi Tıp Fakültesi Göğüs Hastalıkları AD, İstanbul

Dr Ali Kadri Çırak, İzmir Suat Seren Göğüs Hastalıkları ve Göğüs Cerrahisi Eğitim ve Araştırma Hastanesi, İzmir

Dr Serpil Tekgül, İzmir Suat Seren Göğüs Hastalıkları ve Göğüs Cerrahisi Eğitim ve Araştırma Hastanesi, İzmir

Dr Füsun Yıldız, Kocaeli Üniversitesi Tıp Fakültesi, Göğüs Hastalıkları AD, Kocaeli

Dr Berrin Ceyhan, Marmara Üniversitesi Tıp Fakültesi, Göğüs Hastalıkları AD, İstanbul

Dr Sibel Atış Naycı, Marmara Üniversitesi Tıp Fakültesi, Göğüs Hastalıkları AD, İstanbul

Dr Eylem Sercan, Mersin Üniversitesi Tıp Fakültesi Göğüs Hastalıkları AD, Mersin

Dr Tülin Kuyucu, Süreyyapaşa Göğüs Hastalıkları ve Göğüs Cerrahisi Eğitim ve Araştırma Hastanesi, İstanbul

Dr Armağan Hazar, Süreyyapaşa Göğüs Hastalıkları ve Göğüs Cerrahisi Eğitim ve Araştırma Hastanesi, İstanbul

Dr Füsun Şahin, Yedikule Göğüs Hastalıkları ve Göğüs Cerrahisi Eğitim ve Araştırma Hastanesi, İstanbul

Dr Ayşe Bahadır, Yedikule Göğüs Hastalıkları ve Göğüs Cerrahisi Eğitim ve Araştırma Hastanesi, İstanbul

Dr Erdoğan Çetinkaya, Yedikule Göğüs Hastalıkları ve Göğüs Cerrahisi Eğitim ve Araştırma Hastanesi, İstanbul

Dr Müge Meltem Tor, Zonguldak Karaelmas Üniversitesi Tıp Fakültesi Göğüs Hastalıkları AD, Zonguldak

**UNITED KINGDOM**

**England**

Dr Tobenna Onyirioha, Great Western Hospitals NHS Foundation Trust, Swindon

Dr Andrew Stanton, Great Western Hospitals NHS Foundation Trust, Swindon

Dr Alaisdair Stewart, Medway NHS Foundation Trust, Gillingham

Dr Robert Stone, Taunton & Somerset NHS Foundation Trust, Taunton

Ms Tendai Chitakasha, Cambridge University Hosps NHS Foundation Trust, Addenbrookes Hospital, Cambridge

Dr Sian Stinchcombe, Cambridge University Hosps NHS Foundation Trust, Addenbrookes Hospital, Cambridge

Ms Lynn Greatley, Airedale NHS Trust, Airedale General Hospital, Keighley

Dr Vinod Palissery, Airedale NHS Trust, Airedale General Hospital, Keighley

Dr Justin Tuggey, Airedale NHS Trust, Airedale General Hospital, Keighley

Dr Ash Husain, Barnet & Chase Farm Hospitals NHS Trust, Barnet Hospital, Barnet

Dr Rama Vancheeswaran, Barnet & Chase Farm Hospitals NHS Trust, Barnet Hospital, Barnet

Dr Richard Budd, Barnsley Hospital NHS Foundation Trust, Barnsley Hospital, Barnsley

Dr Hazim Mahdi, Barnsley Hospital NHS Foundation Trust, Barnsley Hospital, Barnsley

Dr Nandini Banerjee, Basildon and Thurrock Uni Hsp NHS Foundation Trust, Basildon University Hospital, Basildon

Ms Helen Hill, Basildon and Thurrock Uni Hsp NHS Foundation Trust, Basildon University Hospital, Basildon

Dr Dipak Mukherjee, Basildon and Thurrock Uni Hsp NHS Foundation Trust, Basildon University Hospital, Basildon

Dr Mohammed Azher, Bedford Hospital NHS Trust, Bedford Hospital, Bedford

Ms Lorraine Curtin, Bedford Hospital NHS Trust, Bedford Hospital, Bedford

Dr Enson Thomas, Bedford Hospital NHS Trust, Bedford Hospital, Bedford

Mrs T. Lightowler, Bradford Teaching Hospitals NHS Foundation Trust, Bradford Royal Infirmary, Bradford

Mr Andy O'Dwyer, Bradford Teaching Hospitals NHS Foundation Trust, Bradford Royal Infirmary, Bradford

Dr Katrina Curtis, United Bristol Healthcare NHS Trust, Bristol Royal Infirmary, Bristol

Mr Stuart Metcalfe, United Bristol Healthcare NHS Trust, Bristol Royal Infirmary, Bristol

Dr Jack Kastelik, Hull and East Yorkshire Hospitals NHS Trust, Castle Hill Hospital, Cottingham

Dr David Adeboyeku, The North West London Hospitals NHS Trust, Central Middlesex Hospital, London

Dr Dilys Lai, Chelsea and Westminster Hosp NHS Foundation Trust, Chelsea and Westminster Hospital, London

Dr Martin Allen, University Hospital of North Staffs NHS Trust, City General Hospital, Stoke-on-Trent

Ms Victoria Campbell, University Hospital of North Staffs NHS Trust, City General Hospital, Stoke-on-Trent

Ms Amelia Hilton, Sandwell & West Birmingham Hospitals NHS Trust, City Hospital, Birmingham

Mr George Absi, East Sussex Hospitals NHS Trust, Conquest Hospital, St Leonards-on-Sea

Ms Geraldine Falconer, East Sussex Hospitals NHS Trust, Conquest Hospital, St Leonards-on-Sea

Dr Aravind Ponnuswamy, East Sussex Hospitals NHS Trust, Conquest Hospital, St Leonards-on-Sea

Ms Martina Timon, East Sussex Hospitals NHS Trust, Conquest Hospital, St Leonards-on-Sea

Dr Philip Ryan, Hereford Hospitals NHS Trust, County Hospital, Hereford

Dr Simon Fearby, North Cumbria Acute Hospitals NHS Trust, Cumberland Infirmary, Carlisle

Miss Victoria Lamonby, North Cumbria Acute Hospitals NHS Trust, Cumberland Infirmary, Carlisle

Ms Tendai Zinyengere, Dartford & Gravesham NHS Trust, Darent Valley Hospital, Dartford

Dr Alwyn Foden, County Durham & Darlington NHS Foundation Trust, Darlington Memorial Hospital, Darlington

Dr Kathryn Callaghan, Plymouth Hospitals NHS Trust, Derriford Hospital, Plymouth

Dr Helen Grover, Plymouth Hospitals NHS Trust, Derriford Hospital, Plymouth

Dr Philip Hughes, Plymouth Hospitals NHS Trust, Derriford Hospital, Plymouth

Dr R. Khashkhusha, Mid Yorkshire Hospitals NHS Trust, Dewsbury & District Hospital, Dewsbury

Dr Martin Highcock, Doncaster and Bassetlaw Hosps NHS Foundation Trust, Doncaster Royal Infirmary, Doncaster

Dr Mark Jones, Dorset County Hospital NHS Foundation Trust, Dorset County Hospital, Dorchester

Ms Jayne Manning, Ealing Hospital NHS Trust, Ealing Hospital, Southall

Ms Emma Constantinos, East Sussex Hospitals NHS Trust, Eastbourne District General Hospital, Eastbourne

Dr David Maxwell, East Sussex Hospitals NHS Trust, Eastbourne District General Hospital, Eastbourne

Ms Martina Timon, East Sussex Hospitals NHS Trust, Eastbourne District General Hospital, Eastbourne

Ms Helen Parnell, Epsom & St Helier University Hospitals NHS Trust, Epsom General Hospital, Epsom

Dr Shakil Rahman, Epsom & St Helier University Hospitals NHS Trust, Epsom General Hospital, Epsom

Dr Catherine Houghton, Pennine Acute Hospitals NHS Trust, Fairfield General Hospital, Bury

Dr Tony De Soyza, The Newcastle upon Tyne Hospitals NHSFT, Freeman Hospital, Newcastle Upon Tyne

Dr James Campbell, United Lincolnshire Hospitals NHS Trust, Grantham & District Hospital, Grantham

Dr Anthony Fennerty, Harrogate and District NHS Foundation Trust, Harrogate District Hospital, Harrogate

Dr Robert Buttery, Hinchingbrooke Health Care NHS Trust, Hinchingbrooke Hospital, Huntingdon

Dr Nicky Simler, Hinchingbrooke Health Care NHS Trust, Hinchingbrooke Hospital, Huntingdon

Mr Matthew Hodson, Homerton University Hospital NHS Foundation Trust, Homerton Hospital, London

Dr Nawar Bakerly, Salford Royal NHS Foundation Trust, Hope Hospital, Salford

Dr Jaya Sanganakal, Salford Royal NHS Foundation Trust, Hope Hospital, Salford

Ms Chedia Varden, Salford Royal NHS Foundation Trust, Hope Hospital, Salford

Dr Edward McKeown, Oxford Radcliffe Hospitals NHS Trust, Horton Hospital, Banbury

Dr Annika Graham, Calderdale & Huddersfield NHS Foundation Trust, Huddersfield Royal Infirmary, Huddersfield

Dr Jonathan Douse, Ipswich Hospital NHS Trust, Ipswich Hospital, Ipswich

Ms Claire Chalklin, Maidstone and Tunbridge Wells NHS Trust, Kent & Sussex Hospital, Tunbridge Wells

Ms Sarah Greenslade, Maidstone and Tunbridge Wells NHS Trust, Kent & Sussex Hospital, Tunbridge Wells

Mrs Frances Guyatt, Maidstone and Tunbridge Wells NHS Trust, Kent & Sussex Hospital, Tunbridge Wells

Ms Naomi Hillier, Maidstone and Tunbridge Wells NHS Trust, Kent & Sussex Hospital, Tunbridge Wells

Ms Louise Robertson, Maidstone and Tunbridge Wells NHS Trust, Kent & Sussex Hospital, Tunbridge Wells

Dr Simon Webster, Maidstone and Tunbridge Wells NHS Trust, Kent & Sussex Hospital, Tunbridge Wells

Dr Syed Fayyaz Hussain, Kettering General Hospital NHS Trust, Kettering General Hospital, Kettering

Mr Simon Lee, Kettering General Hospital NHS Trust, Kettering General Hospital, Kettering

Dr Richard Russell, Heatherwood and Wexham Park Hospitals NHSFT, King Edward VII Hospital, Windsor

Ms Jacqui Fenton, Kings College Hospital NHS Foundation Trust, Kings College Hospital, London

Mr Kudzai Mangwende, Kings College Hospital NHS Foundation Trust, Kings College Hospital, London

Dr Michelle Le Cheminant, East and North Hertfordshire NHS Trust, Lister Hospital, Stevenage

Dr Thida Win, East and North Hertfordshire NHS Trust, Lister Hospital, Stevenage

Ms Kathryn Coleman, Maidstone and Tunbridge Wells NHS Trust, Maidstone Hospital, Maidstone

Ms Karen Gardiner, Maidstone and Tunbridge Wells NHS Trust, Maidstone Hospital, Maidstone

Dr Ravish Mankragod, Maidstone and Tunbridge Wells NHS Trust, Maidstone Hospital, Maidstone

Dr Sarah Haines, Cent Manchester/Manchester Chlds Univ Hosp NHST, Manchester Royal Infirmary, Manchester

Dr Shane O'Reilly, Cent Manchester/Manchester Chlds Univ Hosp NHST, Manchester Royal Infirmary, Manchester

Dr Jon Simpson, Cent Manchester/Manchester Chlds Univ Hosp NHST, Manchester Royal Infirmary, Manchester

Dr Shahid Nadeem, Walsall Hospitals NHS Trust, Manor Hospital, Walsall

Dr Milan Bhattacharya, Milton Keynes Hospital NHS Foundation Trus,t Milton Keynes General Hospital, Milton Keynes

Mrs Sandra Olive, Norfolk and Norwich University Hospital NHS Trust, Norfolk and Norwich University Hospital, Norwich

Dr George Hands, Northern Devon Healthcare NHS Trust, North Devon District Hospital, Barnstaple

Dr Alison Moody, Northern Devon Healthcare NHS Trust, North Devon District Hospital, Barnstaple

Dr David Weir, The Pennine Acute Hospitals NHS Trust, North Manchester General Hospital, Manchester

Dr Rachel Tennant, The North West London Hospitals NHS Trust, Northwick Park Hospital, Harrow

Dr Seema Brij, Peterborough & Stamford Hosps NHS Foundation Trust, Peterborough District Hospital, Peterborough

Dr Salim Meghjee, Mid Yorkshire Hospitals NHS Trust, Pinderfields General Hospital, Wakefield

Miss Kathryn Rafferty, Mid Yorkshire Hospitals NHS Trust, Pinderfields General Hospital, Wakefield

Dr Owen Johnson, Mid Yorkshire Hospitals NHS Trust, Pontefract General Infirmary, Pontefract

Ms Jacqui Pollington, Mid Yorkshire Hospitals NHS Trust, Pontefract General Infirmary, Pontefract

Ms Jane Rodger, Mid Yorkshire Hospitals NHS Trust, Pontefract General Infirmary, Pontefract

Ms Sandra Courtiour, Poole Hospital NHS Foundation Trust, Poole Hospital, Poole

Dr Simon Crowther, Poole Hospital NHS Foundation Trust, Poole Hospital, Poole

Dr Ben Green, Portsmouth Hospitals NHS Trust, Queen Alexandra Hospital, Portsmouth

Dr Simon Gompertz, University Hosp Birmingham NHS Foundation Trust, Queen Elizabeth Hospital, Birmingham

Ms Heather Davies, East and North Hertfordshire NHS Trust, Queen Elizabeth II Hospital, Welwyn Garden City

Dr Richard Dent, East and North Hertfordshire NHS Trust, Queen Elizabeth II Hospital, Welwyn Garden City

Dr Shafick Gareeboo, East and North Hertfordshire NHS Trust, Queen Elizabeth II Hospital, Welwyn Garden City

Ms Karen Moore-Haines, East and North Hertfordshire NHS Trust, Queen Elizabeth II Hospital, Welwyn Garden City

Dr Subir Mukherjee, East Kent Hospitals NHS Trust, Queen Elizabeth The Queen Mother Hospital, Margate

Dr Paul Beckett, Burton Hospitals NHS Trust, Queen's Hospital, Burton-on-Trent

Dr Jonathan Corne, Nottingham University Hospitals NHS Trust, Queens Medical Centre Nottingham, Nottingham

Dr Philip Bardsley, The Rotherham NHS Foundation Trust, Rotherham General Hospital, Rotherham

Ms Lauren Bowden, The Rotherham NHS Foundation Trust, Rotherham General Hospital, Rotherham England

Ms Vivenne McGlashan Royal Berkshire NHS Foundation Trust Royal Berkshire Hospital Reading

Dr Anne McGown, Royal Berkshire NHS Foundation Trust, Royal Berkshire Hospital, Reading

Dr Rosalind Green, East Lancashire Hospitals NHS Trust, Royal Blackburn Hospital, Blackburn

Dr Yin Chey Ong, East Lancashire Hospitals NHS Trust, Royal Blackburn Hospital, Blackburn

Ms Christine Peacock, East Lancashire Hospitals NHS Trust, Royal Blackburn Hospital, Blackburn

Dr Bervin Teo, Royal Cornwall Hospitals Trust, Royal Cornwall Hospital, Truro

Dr Will Elston, Derby Hospitals NHS Foundation Trust, Royal Derby Hospital, Derby

Dr David Halpin, Royal Devon & Exeter NHS Foundation Trust, Royal Devon & Exeter Hospital - Wonford, Exeter

Dr John Hurst, Royal Free Hampstead NHS Trust, Royal Free Hospital, London

Dr Rod Lawson, Sheffield Teaching Hospitals NHS Foundation Trust, Royal Hallamshire Hospital, Sheffield

Dr Alison Grove, Winchester & Eastleigh Healthcare NHS Trust, Royal Hampshire County Hospital, Winchester

Mr Barrie Somerville, Winchester & Eastleigh Healthcare NHS Trust, Royal Hampshire County Hospital, Winchester

Ms Susan Baxter, Lancashire Teaching Hospitals NHS Foundation Trust, Royal Preston Hospital, Preston

Ms Angela Miers, Royal Surrey County Hospital NHS Trust, Royal Surrey County Hospital, Guildford

Dr Mark Jackson, Brighton and Sussex University Hospitals NHS Trust, Royal Sussex County Hospital, Brighton

Dr Jay Suntharalingam Royal United Hospital Bath NHS Trust Royal United Hospital Bath England

Dr Graham Burns, The Newcastle upon Tyne Hospitals NHSFT, Royal Victoria Infirmary, Newcastle Upon Tyne

Dr Mazhar Chaudri, The Dudley Group of Hospitals NHS Trust, Russells Hall Hospital, Dudley

Dr Catherine Thompson, Salisbury NHS Foundation Trust, Salisbury District Hospital, Salisbury

Ms Amelia Hilton, Sandwell & West Birmingham Hospitals NHS Trust, Sandwell General Hospital, West Bromwich

Dr Michael Bone, South Tyneside NHS Foundation Trust, South Tyneside District Hospital, South Shields

Mrs Katherine Austin, Southampton University Hospitals NHS Trust, Southampton General Hospital, Southampton

Dr Simon Bourne, Southampton University Hospitals NHS Trust, Southampton General Hospital, Southampton

Ms Patricia Norman, Southampton University Hospitals NHS Trust, Southampton General Hospital, Southampton

Mr Jonathan Watson, Southampton University Hospitals NHS Trust, Southampton General Hospital, Southampton

Dr Jane Wilkinson, Southampton University Hospitals NHS Trust, Southampton General Hospital, Southampton

Dr Sohail Ansari, Southend University Hospital NHS Foundation Trust, Southend Hospital, Westcliff-on-Sea

Dr Duncan Powrie, Southend University Hospital NHS Foundation Trust, Southend Hospital, Westcliff-on-Sea

Dr James Calvert, North Bristol NHS Trust, Southmead Hospital, Bristol

Ms Carla Swift, Southport & Ormskirk Hospital NHS Trust, Southport & Formby District General Hospital, Southport

Dr Alexander Youzguin, Southport & Ormskirk Hospital NHS Trust, Southport & Formby District General Hospital, Southport

Dr David Simcock, Barts and The London NHS Trust, St Bartholomews Hospital, London

Dr Shanthi Paramothayan, Epsom & St Helier University Hospitals NHS Trust, St Helier Hospital, Carshalton

Ms Helen Parnell, Epsom & St Helier University Hospitals NHS Trust, St Helier Hospital, Carshalton

Dr Veronica Varney, Epsom & St Helier University Hospitals NHS Trust, St Helier Hospital, Carshalton

Dr Doytchin Dimov, The Leeds Teaching Hospitals NHS Trust, St James University Hospital, Leeds

Dr Sarah Elkin, Imperial College Healthcare NHS Trust, St Mary's Hospital, London

Ms Sarah Kearney, Isle of Wight NHS Primary Care Trust, St Mary's Hospital, Newport

Ms Hilary Sklar, Imperial College Healthcare NHS Trust, St Mary's Hospital, London

Dr Michael Wood, Ashford & St Peter's Hospital NHS Trust, St Peter's Hospital, Chertsey

Dr Devapriya Dev, Stockport NHS Foundation Trust, Stepping Hill Hospital, Stockport

Dr Maxine Hardinge, Oxford Radcliffe Hospitals NHS Trust, The Churchill, Oxford

Dr Ian Benton, Countess of Chester Hospital NHS Foundation Trust, The Countess of Chester Hospital, Chester

Dr Sharjeela Tariq, Hull and East Yorkshire Hospitals NHS Trust, The Hull Royal Infirmary, Hull

Dr Anur Guhan, South Tees Hospitals NHS Trust, The James Cook University Hospital, Middlesbrough

Maria Taylor, South Tees Hospitals NHS Trust, The James Cook University Hospital, Middlesbrough

Dr Gemina Doolub, Oxford Radcliffe Hospitals NHS Trust, The John Radcliffe, Oxford

Dr Victoria Tippett, Oxford Radcliffe Hospitals NHS Trust, The John Radcliffe, Oxford

Ms Katerina Vernicos, Oxford Radcliffe Hospitals NHS Trust, The John Radcliffe, Oxford

Dr David Morgan, Royal Bournemouth and Christchurch Hosps NHSFT, The Royal Bournemouth Hospital, Bournemouth

Ms Rachel Hardcastle-Jones, South Devon Healthcare NHS Foundation Trust, Torbay Hospital, Torquay

Ms Kay Kerrigan, Trafford Healthcare NHS Trust, Trafford General Hospital, Manchester

Dr Bernard Leahy, Trafford Healthcare NHS Trust, Trafford General Hospital, Manchester

Dr Lisa Davies, Aintree University Hospitals NHS Foundation Trust, University Hospital Aintree, Liverpool

Dr Imran Hafidz, Aintree University Hospitals NHS Foundation Trust, University Hospital Aintree, Liverpool

Dr Anil Trivedi, North Tees & Hartlepool NHS Foundation Trust, University Hospital of Hartlepool, Hartlepool

Dr Neil Leitch, North Tees & Hartlepool NHS Foundation Trust, University Hospital of North Tees, Stockton-on-Tees

Dr Stephen Crooks, South Warwickshire General Hospitals NHS Trust, Warwick Hospital, Warwick

Dr Bobby Mann, West Middlesex University Hospital NHS Trust, West Middlesex University Hospital, Isleworth

Dr Clare Laroche, West Suffolk Hospital NHS Trust, West Suffolk Hospital, Bury St Edmunds

Mrs Sally Smith, West Suffolk Hospital NHS Trust, West Suffolk Hospital, Bury St Edmunds

Dr Erum Arshad, Heatherwood and Wexham Park Hospitals NHSFT, Wexham Park Hospital, Slough

Dr Simon Quantrill, Whipps Cross University Hospital NHS Trust, Whipps Cross University Hospital, London

Dr Sarah Crook, Whittington Hospital NHS Trust, Whittington Hospital, London

Dr Louise Restrick, Whittington Hospital NHS Trust, Whittington Hospital, London

Dr Alan Shaw, Whittington Hospital NHS Trust, Whittington Hospital, London

Ms Sarah Johnson, Worcestershire Acute Hospitals NHS Trust, Worcestershire Royal Hospital, Worcester

Dr Stephen O'Hickey, Worcestershire Acute Hospitals NHS Trust, Worcestershire Royal Hospital, Worcester

Dr Jo Congleton, Western Sussex Hospitals NHS Trust, Worthing and Southlands Hospitals, Worthing

Ms Karen Whittingham, Western Sussex Hospitals NHS Trust, Worthing and Southlands Hospitals, Worthing

Dr Caroline Everett, York Hospitals NHS Foundation Trust, York Hospital, York

Ms Rebecca Sherrington, States of Guernsey Health & Social Services, Princess Elizabeth Hospital, St Martin's

Dr Martin Kelly, Western Health & Social Care Trust, Altnagelvin Area Hospital, Londonderry

Ms Anne-Marie Marley, Belfast Health & Social Care Trust, Belfast City Hospital,Belfast

Ms Rosemary McCoubrey, South Eastern Health & Social Care Trust, Downe Hospital, Downpatrick

Dr Terry McManus, Western Health & Social Care Trust, Erne Hospital, Enniskillen

Dr Lawrence Adu-Boateng, Belfast Health & Social Care Trust, Royal Victoria Hospital, Belfast

Mrs Janet Sinerton, South Eastern Health & Social Care Trust, The Ulster Hospital, Belfast

Dr Wendy Anderson, Northern Health & Social Care Trust, Antrim Hospital, Antrim

Dr Hans-Joerg Hartung, Crosshouse Hospital, Kilmarnock

Prof. William MacNee, Royal Infirmary of Edinburgh, Edinburgh

Ms Kim Bracher, St John’s Hospital at Howden, Livingston

Dr Donald Noble, St John’s Hospital at Howden, Livingston

Dr Fraser Wood, Stirling Royal Infirmary, Stirling

Dr Christine Bucknall, Stobhill General Hospital, Glasgow

Dr Alison Falconer, Stobhill General Hospital, Glasgow

Dr David Sword, The Ayr Hospital, Ayr

Dr Sandra Watson, Wishaw General Hospital, Wishaw

Dr Mark Cotton, Glasgow Royal Infirmary, Glasgow

Dr David Anderson, Victoria Infirmary, Glasgow

Dr Peter Reid, Western General Hospital, Edinburgh

Dr Ramsey Sabit, Cardiff and Vale NHS Trust, Llandough Hospital, Penarth

Ms Rhiannon Skilton, Bro Morgannwg NHS Trust, Neath Port Talbot Hospital, Neath Port Talbot

Dr David Vardill, Bro Morgannwg NHS Trust, Neath Port Talbot Hospital, Neath Port Talbot

Dr Martin Sevenoaks, Bro Morgannwg NHS Trust, Princess of Wales Hospital, Bridgend

Dr Ghulam Shabir, North West Wales NHS Trust, Ysbyty Gwynedd, Bangor

Mrs Sharon Ragbetli, Swansea NHS Trust, Morriston Hospital, Swansea

Dr Madhukar Shetty, Swansea NHS Trust, Morriston Hospital, Swansea

Dr Carol Llewellyn-Jones, Carmarthenshire NHS Trust, West Wales General Hospital, Carmarthen
